# HERC3 regulates epithelial-mesenchymal transition by directly ubiquitination degradation EIF5A2 and inhibits metastasis of colorectal cancer

**DOI:** 10.1038/s41419-022-04511-7

**Published:** 2022-01-21

**Authors:** Zhiyuan Zhang, Guodong He, Yang Lv, Yu Liu, Zhengchuan Niu, Qingyang Feng, Ronggui Hu, Jianmin Xu

**Affiliations:** 1 Department of General Surgery, Zhongshan Hospital, Fudan University, 200030 Shanghai, China; 2 State Key Laboratory of Molecular Biology, Shanghai Institute of Biochemistry and Cell Biology, Center for Excellence in Molecular Cell Science, Chinese Academy of Sciences, 200030 Shanghai, China

**Keywords:** Ubiquitins, Gastrointestinal cancer

## Abstract

E3 ligase is widely reported to exert fundamental functions in cancers. Through rigorous bioinformatic analysis concentrating E3 ligases based on data from Genotype-Tissue Expression (GTEx) and data from The Cancer Genome Atlas (TCGA), HERC3 was indicated to be downregulated in colorectal cancer (CRC) and HERC3 downregulation showed poor overall survival (OS) and disease-free survival (DFS). Through qRT-PCR, western blotting and Immunohistochemistry (IHC), analytical results were validated based on tissues in Zhongshan hospital. Functionally, HERC3 was indicated to inhibit the migration, invasion and metastasis in vitro and in vivo through transwell assays, wound healing assays and vivo experiments. And HERC3 could regulate epithelial-mesenchymal transition (EMT) in CRC. Furthermore, immunoprecipitation (IP), coimmunoprecipitation (co-IP) and GST-pulldown assays indicated that HERC3 could directly interact with EIF5A2 in vitro and in vivo through the RCC1 domain in HERC3. And HERC3 could function as an E3 to promote the K27 and K48-linked ubiquitination degradation of EIF5A2 via the HECT domain in HERC3, besides, K47, K67, K85, and K121 in EIF5A2 were identified as ubiquitination sites. In addition, HERC3 was indicated to affect the migration, invasion and metastasis and further regulatE EMT via EIF5A2/TGF-/Smad2/3 signal. The present study may provide insight into the mechanism of EMT in CRC.

## Introduction

Colorectal cancer (CRC) is a kind of common cancer worldwide [1]. The morbidity and mortality of CRC are raising, nevertheless, the efficient biomarker for treatment, diagnosis and prognostic prediction is still lack, and the mechanism of CRC is still not clarified. Thus, it is urgent to elucidate the potential mechanism of CRC and find effective biomarkers to favor the therapy of CRC.

Ubiquitin proteasome system (UPS) was identified as an important regulator in many biological processes. One crucial effect of UPS is that it can ubiquitinate modify the functional proteins. The process often functions to tag proteins for ubiquitination in a timely and spatially manner. The process is often carried out by ubiquitin ligases (E3s) compounded with accessory adaptors and some other regulatory components. Notably, one critical step in the ubiquitination process is the identification of substrate by E3. The interaction between E3 and substrate is highly specific, and thus E3 is usually recognized as an important role during the process. E3s serve to recruit ubiquitin-loaded E2s, and identify the substrates specifically, finally aid to transfer the ubiquitin to the lysine residues of substrates. The ubiquitination tagged proteins usually go through degradation by proteasomes, however, not all the tagged proteins are degraded, the fate of the proteins is also determined by ubiquitin chain topology. By controlling the ubiquitination of proteins, UPS can govern cell fate decisions such as cell death, cell differentiation, autophagy, and senescence and further exert important functions in regulating the homeostasis, however, the perturbations of UPS especially E3s will also incur many diseases including cancer. Dysregulated E3s can serve as oncogenes or tumor suppressors, furthermore, many oncogenes and tumor suppressors can be regulated by the E3s through ubiquitination degradation. For instance, FBXW7 is commonly identified as a tumor suppressor in various cancers. FBXW7 exerts its function to interact with Skp-Cullin-F-box (SCF) E3s to identify the substrate [2]. Many oncogenes including c-Myc, Notch, mTOR, c-Jun, and Cyclin E can be degraded by SCF FBXW7 through a ubiquitin-mediated, phosphorylation-dependent manner [3–7]. Mutations of Fbw7 are widely identified in diverse cancers including cervical carcinoma, endometrial cancer, and colon cancer [8–10]. Another well-known E3 is MDM2 which is widely observed in many cancers including breast cancer and lung cancer [11, 12]. MDM2 targets many substrates, one of the most famous is the tumor suppressor p53. MDM2 can negatively control the degradation of p53, influence its export from nuclear and affect the translation or transcriptional activity [13–17]. Overexpression of MDM2 results in the degradation of p53 and further promotes the progression of cancers. Moreover, MDM2 may serve as an oncogene independent of p53 [18].

Given the fundamental role of E3s in cancers, we systematically analyzed the dysregulated E3s in CRC based on public databases. Through the rigorous screen, HECT and RLD domain containing E3 ubiquitin-protein ligase 3 (HERC3) was identified as an important E3 in CRC. HERC3 belongs to the HERC E3s family. HERC E3s family contains six E3 ligases that can be further categorized into 2 subgroups based on the protein size including large HERC E3s (HERC1 and HERC2) and small HERC E3s (HERC3, HERC4, HERC5, and HERC6). Many components of the HERC E3s are reported to be crucial in various cancers. HERC1 can modulate breast cancer cells migration and invasion [19]. HERC2 exerts a pivotal function in the p53-MDM2 axis [20]. HERC4 is indicated to promote breast cancer progression via inhibiting tumor suppressor LATS1 via interaction with miRNA [19]. HERC5 is revealed to be a prognostic biomarker for breast cancer through bioinformatic analysis [21]. The relevant research of HERC3 in terms of cancers is rare, HERC3 is once reported to mediate SMAD7 ubiquitination degradation and induce the autophagy-mediated EMT and chemoresistance in glioblastoma [22]. However, research of HERC3 in CRC is still blank.

In this present study. We find that HERC3 is downregulated in CRC and downregulated HERC3 predicts poor prognostic outcomes in terms of overall survival (OS) and disease-free survival (DFS). Moreover, HERC3 was indicated to affect the migration, invasion and metastasis and further regulate EMT via directly ubiquitination degradation EIF5A2. Our study may help to provide insightful views to the treatment of CRC especially in terms of the inhibition of EMT.

## Material and methods

### Acquisition, arrangement of raw data, and data analysis

Corresponding raw data including the expression of normal colonic epithelial tissues from the GTEx and expression of CRC patients’ tissues from TCGA were all obtained from XENA (http://xena.ucsc.edu/). The two individual datasets were normalized and combined according to the description of the relevant datasets. Differential analysis was conducted by the Wilcoxon test. The filter criterion was set as false discovery rate (FDR) < 0.05 and regardless of the value of fold change (Log2FC). The E3s list was obtained from the research [23]. Dysregulated E3s in CRC were systematically analyzed based on The Genotype-Tissue Expression (GTEx) and The Cancer Genome Atlas (TCGA) databases. At first, we combined the E3s expression of normal colon epithelial in the GTEx database and E3s expression of CRC patients’ normal tissues in the TCGA database, and then compared it with the E3s expression of CRC patients’ tumor tissues in the TCGA database and identified the upregulated and downregulated E3s respectively. Secondary, the upregulated and downregulated expressed E3s were also identified respectively by comparing the CRC patients’ normal tissues with CRC patients’ tumor tissues. The intersections of upregulated and downregulated genes were respectively obtained according to the two-step differential analysis. The OS and DFS were visualized in Kaplan-Meier plots and tested by log-rank through R language (Version 3.61). Patients were divided into 2 groups based on the median expression of HERC3. And the prognostic value of dysregulated E3s were also taken into consideration.

### Patients’ tissues

The patients recruited in this study contained two parts. One part included 70 CRC tissues and paired adjacent-normal tissues. These samples were obtained from CRC patients who underwent radical resection in 2019 and were then utilized to detect the expression level of corresponding genes. Another part included 250 CRC tissues, these samples were obtained from patients who underwent radical surgery between 2008 and 2012 and were used to analyze the correlation between the expression of relevant genes and clinical features. All the patients receive nontreatment previous the radical resection surgeries. Informed consent was obtained from patients and all the patients involved in this study were from Zhongshan Hospital, Fudan University. This study was approved by the Clinical Research Ethics Committee of Zhongshan Hospital, Fudan University.

### Cell culture

HEK293T, SW620, DLD-1, SW480 and HCT116 cells were purchased from the American Type Culture Collection (ATCC), and NCM460 cells were purchased from INCELL Corporation. DMEM (Corning) compounded with 10% FBS and penicillin/streptomycin (Life Technologies, USA) was used to culture the cells. Cells were cultured under 5% CO_2_ incubator at 37 °C.

### Construction of stable cell lines and construction, transfection of plasmids

The HERC3 and EIF5A2 overexpression lentivirus (GeneChem, Shanghai, China, HERC3 NCBI reference sequence: NM_014606, EIF5A2 NCBI reference sequence: NM_020390), shHERC3, shEIF5A2 lentivirus and corresponding control lentivirus (GeneChem, Shanghai, China) were obtained. The sequence for shRNAs was as follows: shHERC3: 5′-GGGTGTTATTTGAGAAGTT-3′; shEIF5A2: 5′-GGAUCUUAAACUGCCAGAATT-3′; shNC: 5′-TTCTCCGAACGTGTCACGT-3′. The lentiviruses infected the cells according to the manufacturer’s instructions. The plasmids involved in this study were as follows: pcDNA3.0-HERC3-Flag, pcDNA3.0-EIF5A2-Flag and point mutants, pcDNA3.0-HERC3-Myc and relevant deletion mutants, pcDNA3.0-EIF5A2-Myc, pGEX-4T-1-GST-HERC3, pET22b-EIF5A2-6His, pET28A-His-EIF5A2, pACYC-UBA1-UBCH7-UB-HA-control, and pACYC-UBA1-UBCH7-UB-HA-HERC3 were constructed in our laboratory. For HEK293T cells, polyethylenimine (Sigma-Aldrich, USA) was used to conduct the transfection. And for other cell lines, Lipofectamine 2000 (Life Technologies, USA) was used to conduct the transfection. All the reagents were used according to the manufacturer’s instructions.

### Antibodies

Anti-HERC3 (HPA039170, 1:500 dilution), anti-HA (SAB4300603, 1:5000 dilution), and anti-Myc (SAB4301136, 1:2000 dilution) were purchased from Sigma-Aldrich. Anti-EIF5A2 (16386-1-AP, 1:500 dilution), anti-E-Cadherin (20874-1-AP, 1:5000 dilution), N-Cadherin (22018-1-AP, 1:2000 dilution), Vimentin (10366-1-AP, 1:2000 dilution), anti-GAPDH (60004-1-Ig, 1:2000 dilution), anti-Flag (20543-1-AP, 1:5000 dilution), and anti-GST (HRP-66001, 1:5000 dilution) were purchased from Proteintech. Anti-TGF beta1 (ab215715, 1:1000 dilution), antiphospho-Smad2 (phosphor S467) (ab280888, 1:1000 dilution), antiphospho-Smad3 (phosphor S423 + S425) (ab52903, 1:1000 dilution), anti-Smad2 (ab40855, 1:1000 dilution), and anti-Smad3 (ab40854, 1:1000 dilution) were purchased from abcam. The antibodies were used according to the corresponding instructions.

### qRT-PCR

The total RNA from patients was obtained by RNA simple Total RNA Kit (Tiangen, China) with TRIzol (Invitrogen). Complementary DNA (cDNA) was obtained from total RNA through ReverTra Ace qPCR RT Master Mix (Toyobo, Japan). qRT-PCR was conducted through a 7500 real-time PCR instrument (ABI7500, Thermo Fisher, USA). The expression was all normalized to GAPDH via the 2ΔΔCT method. The primers were as follows: GAPDH forward: 5′-GGAGCGAGATCCCTCCAAAAT-3′, GAPDH reverse: 5′-GGCTGTTGTCATACTTCTCATGG-3′, HERC3 forward: 5′-CTCTGGCAGATCAGCATATCATT-3′, HERC3 reverse: 5′-CAGCTTTTGTATTAACCTGGGCA-3′.

### Western blotting

Samples were all denatured in 2 × sodium dodecyl sulfate-polyacrylamide gel electrophoresis (SDS-PAGE) loading buffer at 100 °C for 15 min and then were transferred into PVDF membranes (Bio-Rad, Shanghai, China). The membranes were incubated with the relevant antibodies sequentially. The signals were imaged by a Tanon 5200 Imaging System (Tanon, China).

### Immunoprecipitation (IP)

Cells were lysed by the RIPA buffer (50 mM Tris-HCl, 5 mM EDTA, 150 mM NaCl, 0.1% SDS, 1% NP-40, and 0.5% sodium deoxycholate, pH 7.6) compounded with protease inhibitor cocktail (Roche). The relevant antibodies and Protein G agarose beads or Flag affinity gels (Sigma-Aldrich, USA, #A4596) were incubated together with the cell lysates at 4 °C overnight.

### Coimmunoprecipitation (co-IP)

Co-IP buffer (50 mM Tris-HCl, 5 mM EDTA, 150 mM NaCl, and 1% NP-40 pH 7.6) mixed with protease inhibitor cocktail (Roche, Switzerland) were used to lyse the cells. The samples were then incubated with the corresponding antibodies and Protein G agarose beads or affinity gels at 4 °C overnight with or without RNase (50 μg/ml).

### Immunohistochemistry (IHC)

All samples were fixed in 4% formalin and embedded in paraffin. The samples were all sliced into 4-μm-thick sections. After further dehydration, peroxidase blocking, and incubation with the designated antibody for 30 min at room temperature. IHC was performed by EnVision Detection Systems Peroxidase/DAB Kit (DAKO, Santa Clara, USA). The intensity of stained cells was scored and classified into 4 grades: grade 0 (no staining), grade 1 (weak staining), grade 2 (intermediate staining), and grade 3 (strong staining). The proportion of positive cells multiplied by the respective intensity cell scores was used as the final staining score (a minimum value of 0 and a maximum of 300). The scoring was done by 2 experts in the field, a third expert would be asked to evaluate if disagreement occurred, and all experts were blinded to the experimental content.

### Immunofluorescence (IF)

Cells were seeded and then were fixed under 4% paraformaldehyde for 20 min, and treated with 0.1% Triton X-100 for 4 h under room temperature. Block buffer (Beyotime, Shanghai, China) was used to block the cells and cells were washed by PBS 3 times. Cells were then incubated with corresponding primary antibodies at 4 °C overnight. And then were incubated with corresponding secondary antibodies for 60 min at room temperature. The cell nuclei were stained by DAPI (Beyotime, Shanghai, China) for 10 min. Fluorescence confocal images were pictured by confocal fluorescence microscopy (Zeiss Germany, Germany)

### Identification of HERC3-binding proteins

HERC3-Flag plasmids were transfected into HCT116 cells and cells were harvested 48 hours after the transfection. Cell lysates were enriched with anti-Flag affinity gels and further eluted in 8 M urea buffer. And the mass spectrometry analysis was conducted.

SWISS MODEL (https://swissmodel.expasy.org/) was used to predict the frame of HERC3 and EIF5A2. The binding sites between HERC3 and EIF5A2 were predicted by ClusPro.

### Obtain of recombinant proteins

Relevant proteins with corresponding tags were expressed through BL21 *Escherichia coli* cells. Cells were then induced by isopropyl β-D-thiogalactoside (IPTG, Sigma-Aldrich, USA) and further pelleted, lysed, and incubated with glutathione or Ni^2+^ nitrilotriacetic acid (NTA) beads to enrich the proteins. 20 mM reduced glutathione (GSH, Sigma-Aldrich, USA) or 250 mM imidazole that was dissolved in PBS buffer (pH 8.0) and was further utilized to wash the products. PBS buffer added with 20% glycerol was used to dialyze the products. The recombinant proteins were stored at −80 °C.

### GST-pulldown assay

The purified corresponding recombinant proteins (20 μg) were mixed together with glutathione sepharose 4B and incubated in 500 μl of pulldown buffer (20 mM Tris-Cl, 5 mM MgCl_2_, 100 mM NaCl, 1 mM DTT, 1 mM EDTA, 0.5% NP-40, and 10 μg/ml BSA pH 7.5). The beads were washed five times by pulldown buffer then were denatured at 100 °C for 15 min in 2× SDS-PAGE loading buffer and further subjected to western blotting.

### In vivo ubiquitylation assay and cycloheximide analysis

Cells were transfected with relevant plasmids and harvested after 48 hours with MG132 pre-treatment. Cells were lysed and proceeded to IP as mentioned above. After incubation, RIPA buffer was used to wash the Flag affinity gels. The gels were then denatured in 2× SDS-PAGE loading buffer at 100 °C for 15 min and the products were then subjected to western blotting. In terms of cycloheximide analysis (CHX-chase assays), cells were treated with CHX (100 µg/ml) and then were harvested at the indicated time and subjected to western blotting.

### Mass spectrometry analysis of ubiquitination modification sites

To identify the ubiquitination modification sites, in vitro ubiquitination assay was conducted. BL21 *E. coli* cells were cotransfected with pET28A-His-EIF5A2 and pACYC-UBA1-UBCH7-UB-HA-control or pACYC-UBA1-UBCH7-UB-HA-HERC3. And cells were further cultured under the culture medium containing ampicillin and chloramphenicol antibiotics. IPTG was incubated with cells and cells were then lysed under 8 M urea lysis buffer. Ni2+ NTA agarose beads and anti-HA affinity gel (Sigma-Aldrich, USA, #E6779) were sequentially used to precipitate the products. Samples were dissolved in 8 M urea and 100 mm Tris-Cl (pH 8.5) and then were proceeded to tris (2-carboxyethyl) phosphine (TCEP) reduction, N-ethylmaleimide (NEM) alkylation and trypsin digestion. The EASY-nLC system (Thermo Fisher, USA) was used to separate the peptides, and the results were analyzed by Thermo Proteome Discoverer 2.1 (Thermo Fisher, USA), and the UniProt Human database (http://www.uniprot.org/) was used as a reference.

### Wound healing assays

Cells were cultured until reaching almost 100% confluency and were seeded to every well of six-well plates with the intensity of 4 × 10^5^ cells/well. A 20 μL pipette tip was used to obtain the linear wound. The serum-free DMEM was used to culture the cells after the scratch. Inverted microscopy was used to detect the migration of cells.

### Transwell assays

With or without 8 μm pore size Matrigel coating (Millipore), transwell assays were carried out to detect the CRC cell migration and invasion. Cells with the intensity of 3 × 10^4^ cells/well were seeded into a single upper chamber under the condition of serum-free DMEM. And 600 μL DMEM compounded with 10% FBS was added into the lower chamber. CRC Cells were cultured at 37 °C for 24 h and then were fixed in 4% paraformaldehyde and further stained with crystal violet dye. Cells inner the chamber were cleansed with a cotton swab. Through microscopy, three fields of the chamber were randomly selected and counted.

### Vivo Experiments

Male BALB/c nude mice aged 5 weeks were purchased Shanghai SLAC Laboratory Animal Co. Ltd. Mice were randomly grouped and were subjected to further assays. By inhalation of 0.5–1.0% isoflurane, mice were anesthetized. The spleen was exposed by opening an incision under the ribs and on the left abdominal wall. Corresponding CRC cells were injected into the distal margin of the spleen. The splenic vessels were ligated, and the spleen was removed. The inner wound and the incision were closed. Mice were sacrificed 6 weeks after injection, and the livers with metastasis were dissected and fixed in paraffin. The study was approved by the Animal Ethics Committee of Zhongshan Hospital Fudan University. Each group had at least 3 mice.

### Statistics

Differentially expressed genes were obtained as mentioned above. Chi-squared test was performed to analyze the correlation between the expression of relevant proteins and clinical characteristics. Pearson correlation was carried out to analyze the expressional correlation between different proteins. Student’s *t*-tests were used to analyze the relevant data, and the results were visualized with GraphPad Prism (GraphPad Software).

## Results

### HERC3 is identified to exert an important role in CRC through bioinformatics analysis

**Fig. 1:**
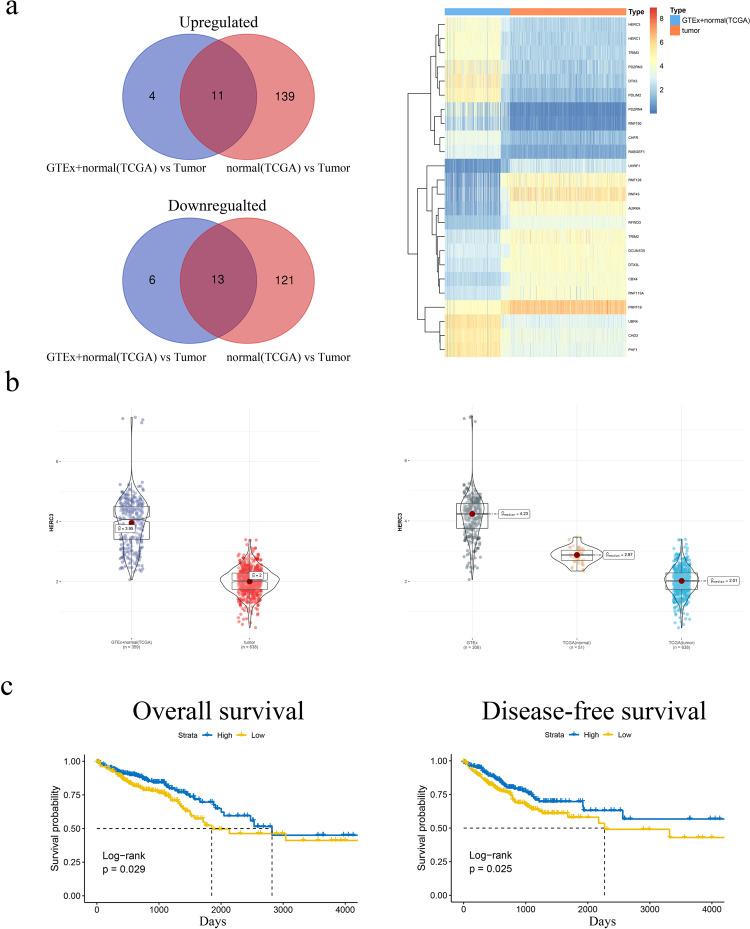
HERC3 is identified to exert an important role in CRC through bioinformatics analysis. **a** Venn plots showed the overlap of the upregulated and downregulated E3s based on differential analysis respectively (left panel). Heatmap showed the expression pattern of the 24 differentially expressed E3s in diverse datasets. **b** Expression pattern of HERC3 in TCGA CRC tissues and in the dataset that was compounded by colon epithelial tissues from GTEx and CRC normal tissues from TCGA (left panel). Expression pattern of HERC3 in colonic epithelial tissues from GTEx, CRC patient adjacent-normal tissues and CRC patient tumor tissues from TCGA (right panel). **c** Overall survival (OS) and disease-free survival (DFS) analysis in CRC patients from TCGA based on the expression of HERC3. Patients were divided into two groups according to the median expression of HERC3. Survival analysis were visualized by Kaplan-Meier plots and tested by log-rank. *P* < 0.05 was identified as statistically significant.

Rigorous analysis that was described in Material and methods were conducted to identify the crucial E3s in CRC. 11 upregulated and 13 downregulated E3s were identified as overlapped according to the two times differential analysis (Fig. 1a, left panel). And the expression pattern of 24 differentially expressed E3s was shown as a heatmap (Fig. 1a right panel). When combing colon epithelial tissues from GTEx and CRC normal tissues from TCGA together, the expression of HERC3 decreased a lot in CRC tumor tissues from TCGA compared with the former merged one **(**Fig. 1b, left panel). Interestingly, we also found that the expression of HERC3 gradually decreased from normal colonic epithelial tissues to CRC patient adjacent-normal tissues to CRC patient tumor tissues (Fig. 1b, right panel). Moreover, when dividing the patients into 2 groups according to the median expression of HERC3, the low expression HERC3 group showed poor OS and DFS (Fig. 1c).

### HERC3 is validated to be downregulated in CRC and downregulated HERC3 predicts poor prognostic outcomes

**Fig. 2:**
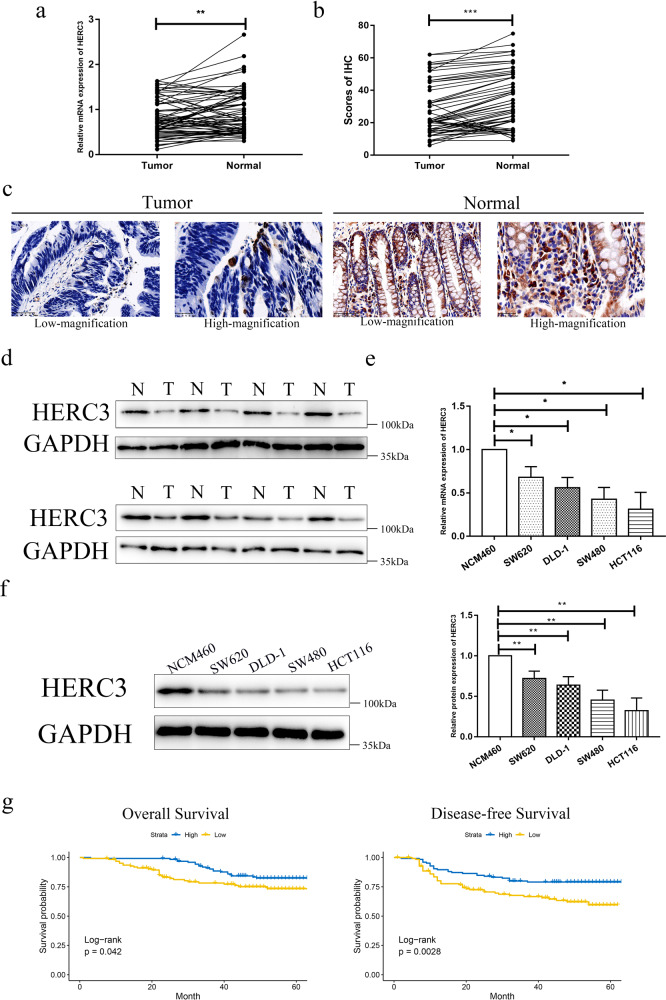
HERC3 is validated to be downregulated in CRC and downregulated HERC3 predicts poor prognostic outcomes. HERC3 was indicated to be downregulated in CRC according to the results of qRT-PCR (**a**) and IHC (**b**) from the 70 paired CRC and adjacent-normal tissues. **c** Representative figures of the expression of HERC3 in paired CRC tumor tissues and adjacent-normal tissues based on the results of IHC, scale bars for low-magnification are 50 μm, for high-magnification are 25 μm. **d** Protein levels of HERC3 in 8 randomly selected paired CRC tissues and CRC adjacent-normal tissues detected by western blotting. **e** The mRNA expression level of HERC3 in NCM460, SW620, DLD-1, SW480, and HCT116 cell lines were detected by qRT-PCR. **f** The protein expression level of HERC3 in NCM460, SW620, DLD-1, SW480, and HCT116 cell lines were detected by western blotting. **g** OS and DFS analysis in 250 patients according to the expression of HERC3, 250 Patients were divided into two groups according to the median expression of HERC3. Survival analysis were visualized by Kaplan-Meier plots and tested by log-rank. *P* < 0.05 was identified as statistically significant.

Based on the results of bioinformatics analysis, we conducted validation in our center. The mRNA expression of HERC3 was downregulated in 70 CRC tumor tissues compared with the paired adjacent-normal tissues (Fig. 2a). Moreover, the protein expression of HERC3 was downregulated in CRC tumor tissues (Fig. 2b, c). We randomly selected 8 paired CRC tumor and adjacent-normal tissues to detect the protein expression of HERC3 through western blotting. Consistently, the protein expression of HERC3 in CRC was downregulated (Fig. 2d). We also detected the expression of HERC3 in several CRC cell lines and NCM460 cell lines, compared with NCM460 cell lines, the mRNA and protein expression of HERC3 all decreased in CRC cell lines (Fig. 2e, f). Based on the median protein expression of HERC3 in CRC tissues, 250 CRC patients were classified into 2 groups. Expression of HERC3 was revealed to be associated with T, N, and M stage (Table 1). Moreover, the HERC3 downregulation group showed poor clinical outcomes in terms of OS and DFS (Fig. 2g).

**Table 1:** Correlation between clinical features and expression of HERC3.

Characteristics	Number	HERC3 expression		*P*-value
		High	Low	
T stage				0.03
T1 + T2	37	25	12	
T3 + T4	213	100	113	
N stage				0.0003
≥1	103	37	66	
0	147	88	59	
M stage				0.02
1	50	17	33	
0	200	108	92	

### HERC3 inhibits the migration, invasion and metastasis of CRC via regulating EMT

**Fig. 3:**
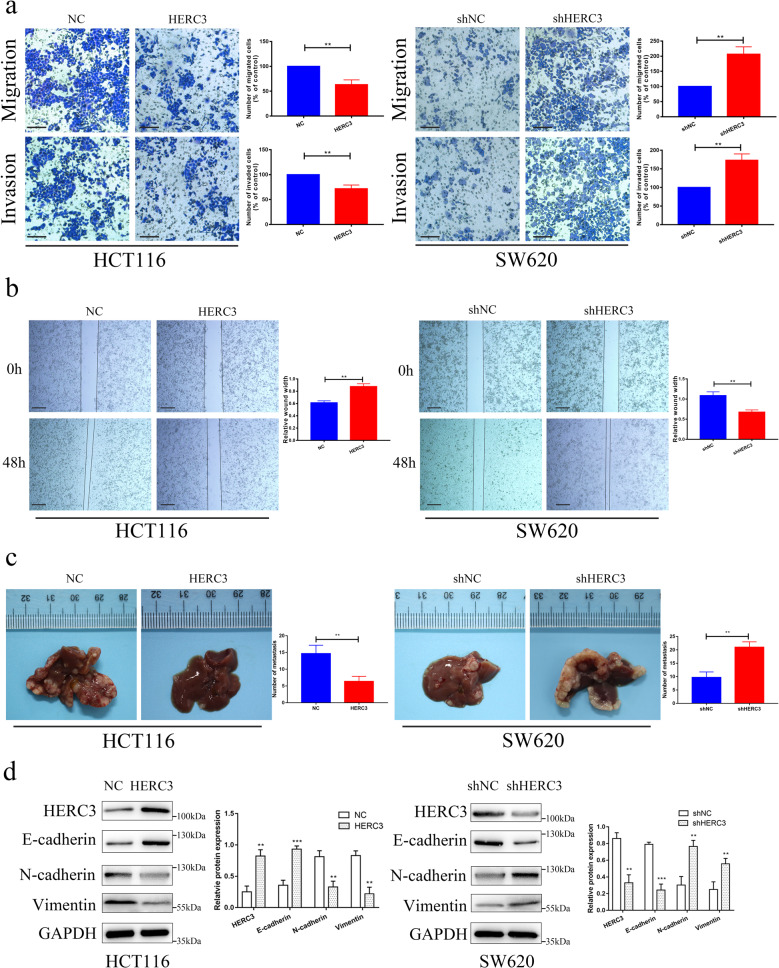
HERC3 inhibits the migration, invasion and metastasis of CRC via regulating EMT. **a** Transwell assays (**a**) and wound healing assays (**b**) indicated that HERC3 upregulation could inhibit the migration and invasion in HCT116 cells while HERC3 downregulation could increase migration and invasion in SW620 cells. Scale bar for transwell assays was 100 μm. Scale bar for wound healing was 250 μm. **c** HERC3 upregulation could inhibit CRC metastasis and HERC3 downregulation could increase CRC metastasis in vivo with corresponding stable cell lines. **d** Western blotting showed that HERC3 upregulation increased the expression of E-cadherin, and decreased expression of N-cadherin and Vimentin in HCT116 cell lines. And HERC3 downregulation decreased the E-cadherin expression and increased the N-cadherin and Vimentin expression in SW620 cell lines.

According to the expression of HERC3 in four CRC cell lines, we selected SW620 and HCT116 to construct two stable cell lines. In HCT116 cell lines that showed relatively low expression of HERC3 compared with other CRC cell lines, HERC3 was upregulated, and in SW620 that showed relatively high expression of HERC3, HERC3 was downregulated. The efficiency of the corresponding lentivirus was checked in Supplementary Fig. S1a. As mentioned above, HERC3 was correlated with T, N, and M stage which was associated with the cancer cell invasion, migration and metastasis, further experiments were performed. The results demonstrated that HERC3 inhibited the migration and invasion in HCT116 cells and HERC3 downregulation increased the migration and invasion in SW620 cells (Fig. 3a). Moreover, we found that downregulation of HERC3 increased the migration of SW620 cells and upregulation HERC3 inhibited the migration of HCT116 cells through wound healing assays (Fig. 3b). Moreover, upregulated HERC3 in HCT116 cells inhibited the metastasis of CRC in vivo compared with the control group. And downregulated HERC3 in SW620 cells increased the metastasis of CRC in vivo (Fig. 3c). The hematoxylin–eosin (HE) stain confirmed the metastasis (Supplementary Fig. S1b). Due to the crucial role of EMT in the regulation of metastasis in CRC [24]. We detected the effects of HERC3 on EMT. As shown in Fig. 3d. Upregulation of HERC3 increased the expression of E-cadherin and decreased the expression of N-cadherin and Vimentin in HCT116 cell lines. And downregulation HERC3 decreased the E-cadherin expression and increased the N-cadherin and Vimentin expression in SW620 cell lines (Fig. 3d). The effects of HERC3 on EMT were verified according to samples obtained from the vivo experiments mentioned in this section via western blotting (Supplementary Fig. S1c). Furthermore, the effects of HERC3 on EMT were also validated by IF (Supplementary Fig. S2). Moreover, 70 patients were divided into 2 groups based on the median expression of HERC3 which was detected by IHC, patients who had relatively high expression level of HERC3 showed higher expression of E-cadherin, lower N-cadherin, and lower Vimentin. Patients who had relatively low expression level of HERC3 showed lower expression of E-cadherin, higher N-cadherin, and higher Vimentin (Supplementary Fig. S3). Thus, Clinical samples further validated the results and indicated HERC3 could regulate the EMT.

### Identification and validation of EIF5A2 as a HERC3-interaction protein

**Fig. 4:**
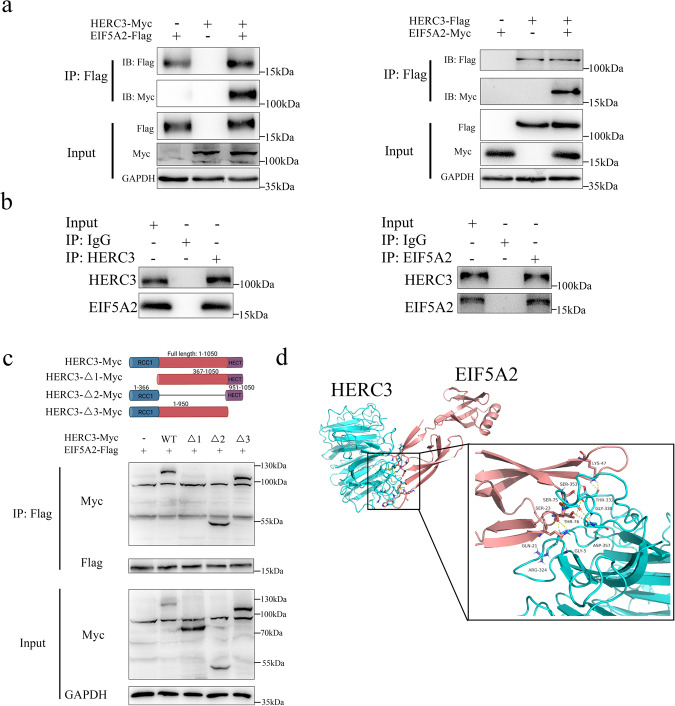
Identification and validation of EIF5A2 as a HERC3-interaction protein. **a** Exogenously expressed EIF5A2 and HERC3 could interact with each other. HCT116 cells were transfected with indicated plasmids and then were subjected to co-IP. **b** Endogenous HERC3 could interact with EIF5A2, the lysates of HCT116 cells were subjected to co-IP with anti-HERC3 or anti-EIF5AH2 and control IgG. **c** The interaction between HERC3 and EIF5A2 depended on the RCC1 domain in HERC3. HCT116 cells were transfected with indicated plasmids and subjected to co-IP. **d** Detailed hydrogen bonding sites between amino acids in HERC3 and EIF5A2. The structure of proteins was visualized by SWISS-MODEL, and the analysis of the interaction was performed by ClusPro.

HCT116 cells were selected due to the relatively low expression level of HERC3 compared with other CRC cell lines which might better imitate the low expression level of HERC3 in CRC tumors. IP and mass spectrometry analysis were conducted to integrate screen the proteins that can interact with HERC3, based on the results of mass spectrometry analysis, EIF5A2 possessed a higher likelihood to interact with HERC3 compared to many other proteins, moreover, EIF5A2 was previously reported to induce EMT in CRC which may better help illustrate the effects of HERC3 on EMT [25]. Thus, we hypothesized that EIF5A2 might be able to interact with HERC3. The liquid chromatography-tandem mass spectrometry (LC-MS/MS) spectra that represented EIF5A2 was shown in Supplementary Fig. S4a. Through co-IP assays, we confirmed that exogenous and endogenous could interact with each other (Fig. 4a, b). Moreover, through co-IP assays, we also found the interaction between HERC3 and EIF5A2 decreased a lot after the deletion of the RCC1 domain in HERC3 that indicating that the interaction mainly depended on the RCC1 domain in HERC3 (Fig. 4c). And the interactions between the RCC1 domain in HERC3 and EIF5A2 were predicted to be several hydrogen bonding sites formed by indicated amino acids that further validated our experiments **(**Fig. 4d). Through GST-pulldown assay, we found that HERC3 could directly interact with EIF5A2 in vitro (Supplementary Fig. S4b). And immunofluorescence assays indicated that HERC3 could colocalize with EIF5A2 in HCT116 cells (Supplementary Fig. S4c)

### EIF5A2 is ubiquitination degraded by HERC3 through HECT domain at multiple sites via a proteasome-dependent way

**Fig. 5:**
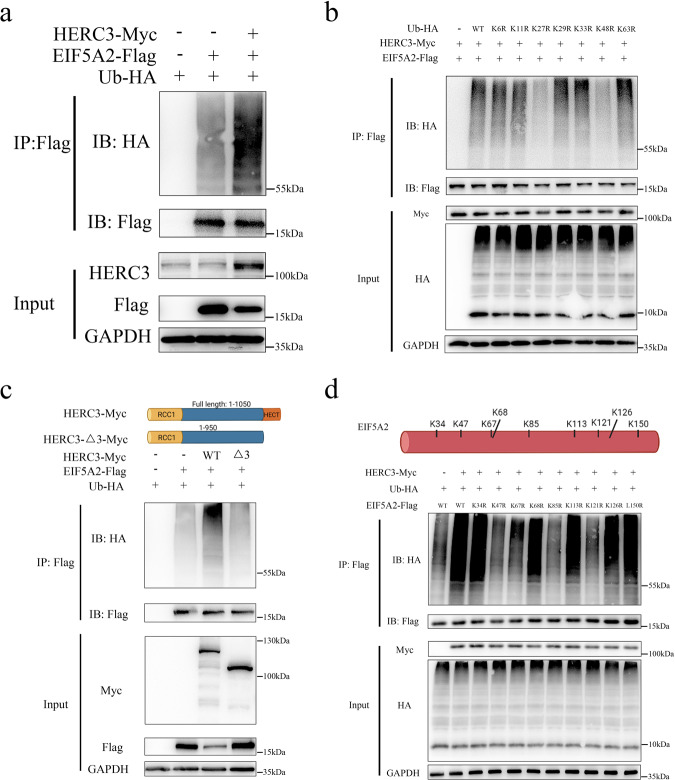
EIF5A2 is ubiquitination degraded by HERC3 through HECT domain at multiple sites via a proteasome-dependent way. **a** HERC3 could promote the ubiquitination of EIF5A2 in HCT116 cells. **b** HERC3 conjugated poly-Ub to EIF5A2 in a K27 and K48 manner, seven HA-Ub mutants bearing K-to-R changes were cotransfected with the relevant plasmids into HEK293T cells and were further subjected to co-IP. **c** Deletion of the HECT domain inhibited the ubiquitination of EIF5A2 significantly. Wild-type HERC3 or deletion mutant (as the schematic diagram shows above) were cotransfected with other indicated plasmids into HEK293T cells and were then subjected to co-IP. **d** K47, K67, K85, and K121 on EIF5A2 were responsible for HERC3-mediated ubiquitylation in HEK293T cells. HEK293T cells were cotransfected with indicated plasmids and then subjected to co-IP.

HERC3 exerts its functions as an E3 in many biological processes, we wondered if EIF5A2 can be ubiquitinated modified by HERC3, in vivo ubiquitylation assay was conducted, and the results indicated that HERC3 promoted the ubiquitination of EIF5A2 in HCT116 cells (Fig. 5a). Moreover, seven HA-Ub mutants bearing K-to-R changes were cotransfected with the corresponding plasmids into HEK293T cells and then subjected to co-IP. The results indicated that HERC3 might conjugate poly-Ub to EIF5A2 in a K27 and K48 manner (Fig. 5b). Vivo ubiquitylation experiments also demonstrated that after the deletion of the HECT domain in HERC3, the ubiquitination of EIF5A2 reduced dramatically in HEK293T cells (Fig. 5c). Through mass spectrometry analysis, nine Lys residues in EIF5A2 were identified as side chains by which HERC3 might conjugate poly-Ub chains (Supplementary Table S1, Supplementary Fig. S5A–P). Vivo ubiquitylation experiments with nine individual EIF5A2 mutants plasmids bearing K-to-R changes confirmed that K47, K67, K85, and K121 on EIF5A2 were responsible for HERC3-mediated ubiquitylation in HEK293T cells (Fig. 5d). Besides, we found that ectopic expression of HERC3 could result in the degradation of EIF5A2 in a dose-dependent manner in HCT116 cells (Supplementary Fig. S6a). We also found that MG132 could restore the degradation induced by HERC3 upregulation and MG132 could increase the protein levels of EIF5A2 in HCT116 cells, indicating that HERC3 could ubiquitinate degradation EIF5A2 in a proteasome-dependent pathway (Supplementary Fig. S6b). CHX-chase assays indicated that HERC3 upregulation could reduce the half-life of EIF5A2 in HCT116 cells (Supplementary Fig. S6c). Moreover, we analyzed the correlation between EIF5A2 and HERC3 in 70 CRC tissues and results indicated that HERC3 was negatively correlated with EIF5A2 (Supplementary Fig. S6d). When dividing the 70 CRC patients into two groups according to the median expression of HERC3, we found that the high HERC3 expression group showed relatively low expression of EIF5A2 and the low HERC3 expression group showed relatively high expression of EIF5A2 (Supplementary Fig. S6e). Besides, according to the median expression of HERC3 and EIF5A2, 250 patients were divided into two groups, patients who had high expression of HERC3 and low expression of EIF5A2 showed better prognostic outcomes than patients with low expression HERC3 and high expression EIF5A2 (Supplementary Fig. S6f). These results all indicated that EIF5A2 was ubiquitination degraded by HERC3 through the HECT domain at multiple sites via a proteasome-dependent way.

### HERC3 regulates the EMT via EIF5A2/TGF-/Smad2/3 signal

Previous results in this research also indicated that HERC3 could regulate the EMT in CRC cells, and HERC3 could promote ubiquitination degradation EIF5A2, we wonder if the effects of HERC3 on EMT are dependent on EIF5A2, further experiments were performed. Through western blotting and IF, first, we found that EIF5A2 downregulation could independently increase the expression of E-cadherin and decrease the expression of N-cadherin and Vimentin in HCT116 cells and EIF5A2 overexpression could decrease the expression of E-cadherin and increase the expression of N-cadherin and Vimentin in SW620 cells. Moreover, EIF5A2 could modulate TGF-/Smad2/3 signal in thyroid carcinoma [26]. And this regulatory still worked in CRC cell lines, EIF5A2 could activate the TGF-β signaling. Overexpression EIF5A2 could enhance the expression of TGF-β1, p-Smad2 and p-Smad3 and downregulation EIF5A2 could inhibit the expression of TGF-β1, p-Smad2 and p-Smad3 (Supplementary Fig. S7a, b). And the effects of EIF5A2 on EMT was also proved via IF (Supplementary Fig. S8).

**Fig. 6:**
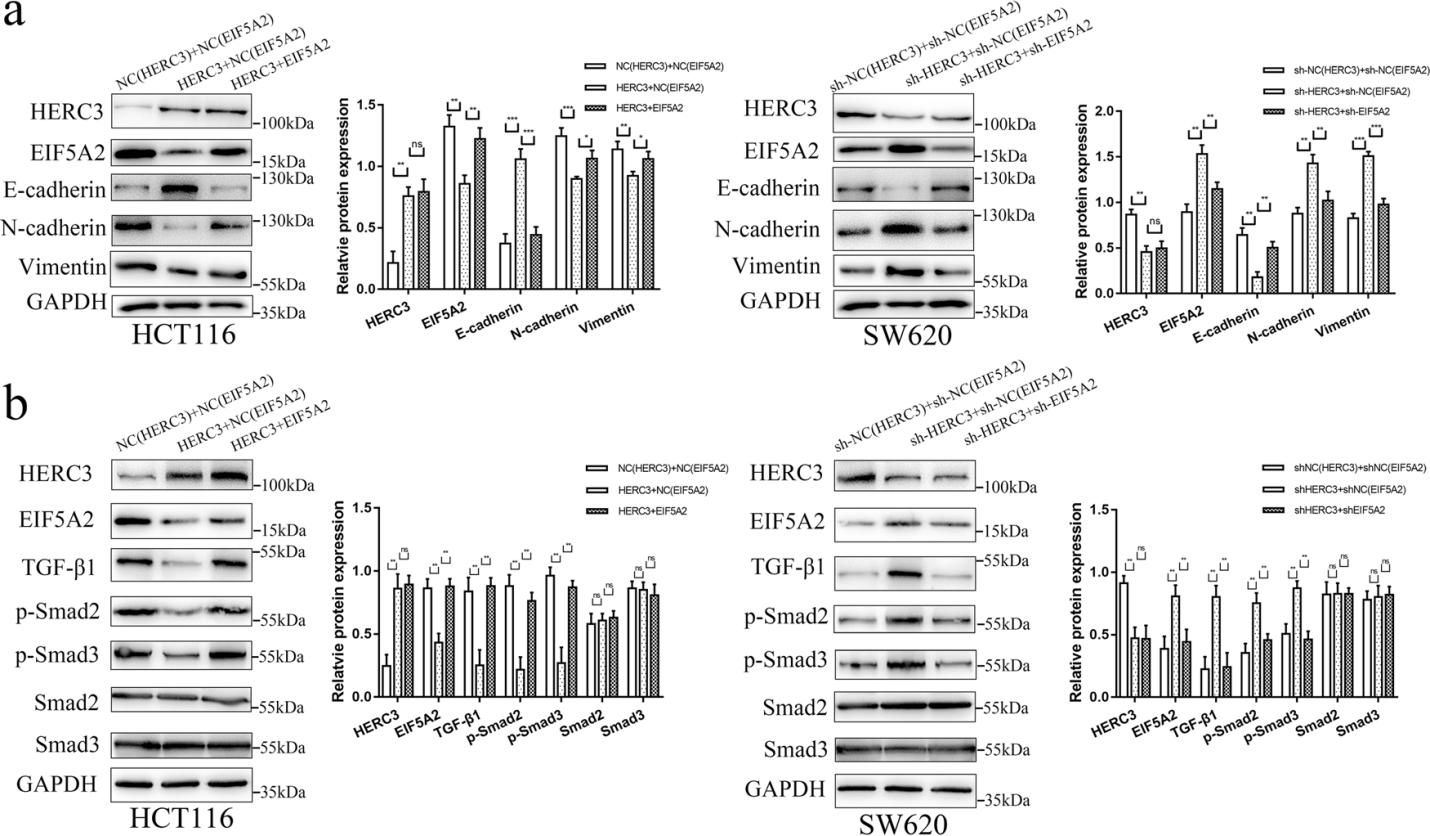
HERC3 regulates the EMT via EIF5A2/TGF-/Smad2/3 signal. Western blotting (**a** and **b**) indicated that EIF5A2 overexpression could rescue the effects of HERC overexpression on EMT and TGF-/Smad2/3 signal in HCT116 cells and EIF5A2 downregulation could also rescue the effects of HERC3 downregulation on EMT and TGF-/Smad2/3 signal in SW620 cells. Experiments were conducted with indicated cell lines.

The efficiency of lentivirus coinfection was confirmed (Fig. 6a). Rescue experiments were carried out and the results of western blotting indicated that EIF5A2 overexpression could rescue the effects on EMT and TGF-/Smad2/3 signal caused by HERC3 overexpression in HCT116 cells and EIF5A2 downregulation could also rescue the effects on EMT and TGF-/Smad2/3 signal induced by HERC3 downregulation in SW620 cells (Fig. 6a, b). And the rescue effects of EIF5A2 on EMT caused by HERC3 were also validated via IF (Supplementary Fig. S9). Taken together, it indicated that HERC3 could regulate EMT through EIF5A2/TGF-/Smad2/3 signal.

### HERC3 inhibits the migration, invasion and metastasis of CRC via EIF5A2

**Fig. 7:**
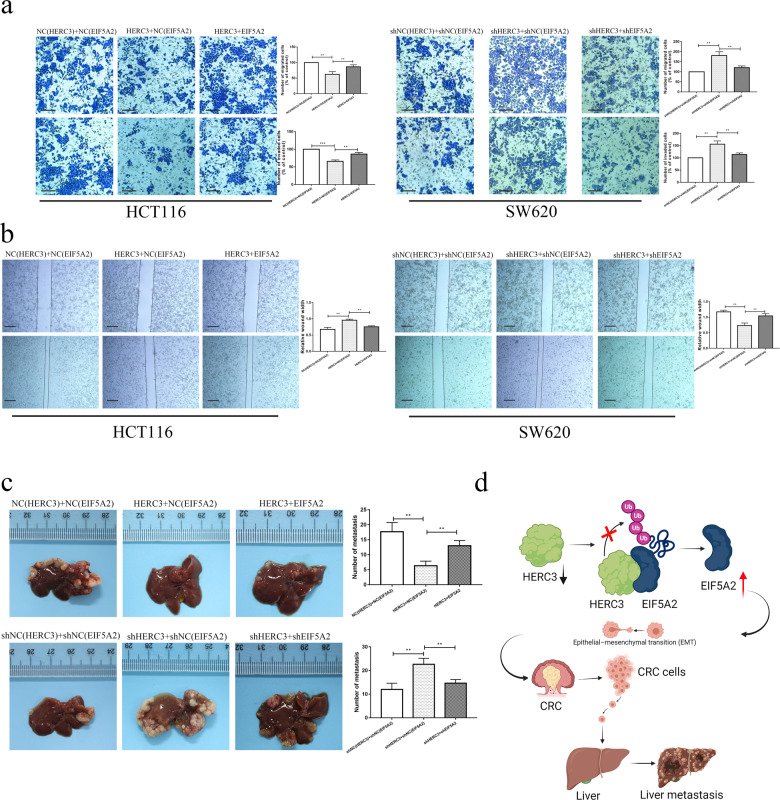
HERC3 inhibits the migration, invasion and metastasis of CRC via EIF5A2. Transwell assays (**a**) and wound healing assays (**b**) indicated that EIF5A2 could attenuate the effects of HERC3 on HCT116 cells and SW620 cells in terms of migration and invasion. Scale bar for transwell assays was 100 μm. Scale bar for wound healing was 250 μm. Vitro experiments were conducted by indicated cell lines and the efficiency of the corresponding lentivirus was confirmed as shown in Fig. 6a. **c** EIF5A2 could also rescue the effects of HERC3 in terms of the metastasis of CRC in vivo. Vivo experiments were conducted with indicated cell lines. **d** Potential regulatory mechanism in this study.

We wondered if the effects of HERC3 on the migration and invasion in CRC is also dependent on EIF5A2. We found that EIF5A2 downregulation could inhibit the migration and invasion of HCT116 cells and EIF5A2 upregulation could increase the migration and invasion of SW620 cells indicating that EIF5A2 could affect the migration and invasion of CRC cells independent of HERC3 (Supplementary Fig. S10a, b). Moreover, through transwell assays and wound healing assays with corresponding cell lines, we found that EIF5A2 could attenuate the effects of HERC3 on HCT116 cells and SW620 cells in terms of cell migration and invasion (Fig. 7a, b). EIF5A2 could also rescue the effects of HERC3 on CRC in terms of metastasis in vivo (Fig. 7c). The metastasis in the liver from vivo experiments was confirmed by HE stains (Supplementary Fig. S11). Taken together, these findings indicated that HERC3 might exert its functions through regulating EIF5A2.

In conclusion, HERC3 could regulate EMT via ubiquitination degradation EIF5A2 and further inhibit the metastasis of CRC. The potential regulatory mechanism is shown in Fig. 7d.

## Discussion

UPS plays important role in even every biological process. Almost every protein will be subjected to ubiquitylation at least once. It’s widely recognized that ubiquitylation is cooperated by ubiquitin-activating enzymes (E1s), ubiquitin-conjugating enzymes (E2s) and ubiquitin ligases (E3s). Among these members in UPS, E3s can be identified as a crucial component because it can specifically recognize the substrates and promote the ubiquitination of the target substrates. E3s involves in many processes to stable homeostasis. In addition, E3s also play critical roles in diseases, including cancers. First, many oncogenes and tumor suppressors can be ubiquitinated modified by E3s, besides, E3s can serve as oncogenes or tumor suppressors in various cancers. For example, CRL3^BTBD9^ degrades TNFAIP1 and further regulates lung cancer metastasis [27]. TRIM7 ubiquitination degrades Src and inhibits liver cancer progression [28]. RNF144A suppresses breast cancer by regulating HSPA2 [29]. Studies focusing on E3s can help to elucidate the potential mechanism of diverse cancers. Moreover, growing evidence implies that dysregulated E3s can also affect the response to remedy and further indicates that E3s can be translated into clinical treatment such as serving as promising therapeutic targets of anticancer drugs.

Given such important roles of E3s in cancers, we comprehensively screened out the dysregulated E3s in CRC through bioinformatics analysis based on public databases. Through rigorous twice differential analysis, 24 dysregulated E3s in CRC were identified. Taken the clinical value of the 24 dysregulated E3s, we speculated HERC3 to exert significant functions in CRC because HERC3 was the only E3, which showed the same trend of differential expression and survival analysis (downregulated in CRC and downregulation indicated poor DFS and OS). Interestingly, HERC3 showed a gradual decrease trend from healthy individual’s colonic epithelial tissues to CRC patients’ tumor-adjacent-normal tissues to CRC patients’ tumor tissues that implied that HERC3 might involve in the whole process of CRC including initiation and progression. HERC3 contains the HECT domain and is one family member of the six HERC E3 family that both have HECT and RCC1-like domain. HERC E3 family consists of six E3s and can be classified into two subgroups based on the protein size. HERC3 is one member of the four small HERC E3s. HERC E3s are previously reported to have pivotal roles in many cancers. For instance, HERC1 was indicated to modulate the migration and invasion in breast cancer cells [19]. HERC2 was reported to have an important function in the p53-MDM2 axis and might provide novel insight in cancers [20]. HERC4 interacts with miRNA and induces breast cancer progression via inhibiting LATS1 [19]. HERC5 is identified to be a potential prognostic biomarker for breast cancer via bioinformatics analysis [21]. However, studies focusing on the functions of HERC3 in cancer is still rare. And to our knowledge, HERC3 is only once reported to promote ubiquitination degradation SMAD7 and induces autophagy-mediated EMT in glioblastoma [22]. However, the study concentrating on the effects of HERC3 in CRC is blank.

The analysis was further confirmed based on tissues in Zhongshan hospital. Through bioinformatics analysis and experimental confirmation, HERC3 was revealed to be downregulated in CRC tissues compared to adjacent-normal tissues, and HERC3 downregulation showed poor prognostic outcomes including DFS and OS. However, some patients also showed high expression of HERC3 in tumor tissues compared to tumor-adjacent tissues. It may be due to the different mechanisms among the development of CRC, perhaps there are other genes not HERC3 that play an important role in the development of disease in this small group of patients, perhaps it may also be due to the different tumor genomic type in this group of patients, resulting in different expression trends of HERC3 from other patients, however, based on the available clinical data, we did not find a specific tumor genomic type for this group of patients, maybe an unidentified one, which is one of the directions of our future research. In addition, HERC3 was indicated to be associated with T, N, and M stage which was associated with the cancer cell migration, invasion, and metastasis, further experiments were performed. Through vitro and vivo experiments, we found that HERC3 could inhibit the CRC cell migration, invasion, and metastasis.

Due to the crucial role of EMT in the regulation of metastasis in CRC [24]. We also researched the effects of HERC3 on EMT in CRC. Excitingly, HERC3 was indicated to influence the expression of several EMT biomarkers in CRC. EMT is a kind of process that epithelial cells are depolarized and achieve the ability to migrate and invade [30]. EMT is an indispensable process for tumor cells to move to adjacent cell layers from the original solid tumors, and can be concluded as a cellular process that enhances the cancer cells migration and invasion [31]. Thus, HERC3 might also play a fundamental role in regulating EMT and further inhibit the metastasis of CRC.

However, how HERC3 regulates the EMT of CRC is still unknown. So further studies were carried out. Through diverse vitro experiments, EIF5A2 was identified as a substrate of HERC3. HERC3 was indicated to promote the ubiquitination degradation of EIF5A2. In addition, the detailed regulatory mechanism between HERC3 and EIF5A2 was elucidated in this study. The RCC1 domain in HERC3 was mainly responsible for the interaction between HERC3 and EIF5A2, and the ubiquitination of EIF5A2 was mainly dependent on HECT domain in HERC3. And the K47, K67, K85, and K121 lysine residues in EIF5A2 were responsible for the ubiquitination modification. Moreover, we also discovered that HERC3-mediacted ubiquitination of EIF5A2 was in the K27 and K48-linkage manner. As commonly recognized, K48-linkage is the classical type of poly-Ub chain that leads to degradation [32]. Moreover, rescue experiments were performed, results indicated that HERC3 influenced the migration, invasion, metastasis and EMT via regulating EIF5A2. EIF5A2 was reported to promote CRC EMT and CRC aggressiveness which might also help to explain the effects of HERC3 on CRC [25]. To our knowledge, our study is the first one to report the effects of HERC3 on CRC in terms of migration, invasion, and metastasis. And we further explained the detailed mechanism as HERC3 affected EMT and functions of CRC cells via ubiquitination degrading EIF5A2. Moreover, the detailed ubiquitination regulatory mechanism of EIF5A2 mediated by HERC3 was also explained as HERC3 promoted the ubiquitination of EIF5A2 through HECT domain at multiple sites in EIF5A2 via a proteasome-dependent way. The detailed ubiquitination regulatory mechanism might help to invent the target antitumor drugs. Moreover, we further researched the potential downstream of EIF5A2 in CRC, we found that HERC3 might regulate the CRC via EIF5A2/TGF-/Smad2/3 signal.

Based on the gradient differential expression trend of HERC3 from normal colorectal epithelial tissues to tumor-adjacent-normal tissues and to CRC tissues, HERC3 may play an important role in the whole process of CRC development. The targeted intervention of HERC3 at the early stage of CRC may reverse the progression of colorectal cancer, and targeted intervention of HERC3 at the progressive stage of CRC may also help in the treatment of CRC, so there is great potential value for the research and subsequent clinical translation of HERC3-targeted drugs. Secondly, due to the prognostic value of HERC3 and the existence of the former mentioned gradient differential expression trend, perhaps HERC3 can be used as an early diagnosis indicator for CRC and also a prognostic biomarker for CRC diagnosis and treatment. However, our study has some limitations, as our clinical sample size is not large enough for the exploration of the prognostic role of HERC3, and we lack normal human colorectal epithelial samples for relevant validation based on the gradient differential expression trend of HERC3.

In conclusion, we gave strong evidence that HERC3 could inhibit migration, invasion and metastasis of CRC by inducing the ubiquitination degradation of EIF5A2 and further regulate the EIF5A2/TGF-/Smad2/3 signal in CRC. Our study may provide a novel insight into the mechanism and treatment of CRC especially in terms of metastasis of CRC.

## Supplementary information

Supplementary Figure and Table legend

Supplementary Fig. S1.

Supplementary Fig. S2.

Supplementary Fig. S3.

Supplementary Fig. S4.

Supplementary Fig. S5.

Supplementary Fig. S6.

Supplementary Fig. S7.

Supplementary Fig. S8.

Supplementary Fig. S9.

Supplementary Fig. S10.

Supplementary Fig. S11.

Supplementary Table S1

Reproducibility checklist

Response of the change of the authors

Change of authorship request form

## Data Availability

The data in the study are available upon reasonable request.

## Code availability

Codes are available upon reasonable request.
