# Supplementary material for: HERC3 regulates epithelial-mesenchymal transition by directly ubiquitination degradation EIF5A2 and inhibits metastasis of colorectal cancer
Source: Cell Death Dis. 2022 Jan 21;13(1):74. doi: 10.1038/s41419-022-04511-7 (PMC8782983; doi:10.1038/s41419-022-04511-7)
Supplement: Supplementary file 1 — Supplementary Figure and Table legend [file 41419_2022_4511_MOESM1_ESM.docx]

**Supplementary Figure legend**

Supplementary Fig. S1. Efficiency of the lentivirus was validated by western blotting in HCT116 cells and SW620 cells (a). Hematoxylin–eosin (HE) stain confirmed the metastasis in the liver formed by indicated cell lines in vivo experiments. Scale bars for low-magnification are 1000μm, for high-magnification are 100μm (b). Western blotting indicated that HERC3 could regulate the EMT in CRC based on samples from the vivo experiments mentioned in Fig 3c (c).

Supplementary Fig. S2. Immunofluorescence (IF) validated the effects of HERC3 on several EMT markers including E-cadherin, N-cadherin, and Vimentin, scale bar for IF was 50μm.

Supplementary Fig. S3. Patients who had relatively high expression level of HERC3 showed higher expression of E-cadherin, lower N-cadherin, and lower Vimentin. Patients who had relatively low expression level of HERC3 showed lower expression of E-cadherin, higher N-cadherin, and higher Vimentin. 70 patients were divided into 2 groups based on the median expression of HERC3 which was detected by IHC.

Supplementary Fig. S4. Representative peptide of EIF5A2 that was identified by mass spectrometry analysis was shown as the liquid chromatography-tandem mass spectrometry (LC-MS/MS) spectra (a). HERC3 could directly bind EIF5A2 in vitro. GST pulldown assays were performed with indicated recombinant proteins and were then detected by western blotting (b). Exogenous HERC3 and EIF5A2 could colocalize in HCT116 cells detected by immunofluorescence staining, scale bars were 50μm (c).

Supplementary Fig. S5. Peptides identified by mass spectrometry analysis for the identification of the ubiquitination modification sites are shown as the liquid chromatography-tandem mass spectrometry (LC-MS/MS) spectra. Figures are labeled by a-p orderly according to the scores in Supplementary Table S1.

Supplementary Fig. S6. Ectopic expression of HERC3 promoted degradation of EIF5A2 in a dose-dependent manner in HCT116 cells (a). EIF5A2 was validated to be degraded in a ubiquitin-proteasome-dependent manner. HCT116 were transfected with indicated plasmids or treated with MG132 and then subjected to IP (b). CHX chase assays indicated that HERC3 overexpression could reduce the EIF5A2 half-life in HCT116 cells (c). The correlation between HERC3 and EIF5A2 was tested by chi-squared test (left panel) and HERC3 was negatively correlated with EIF5A2 based on results of IHC in 70 CRC tissues (right panel) (d). Representative images showed the expression of HERC3 and EIF5A2 in the indicated groups (continuous slices in individual groups and were further subjected to IHC with the indicated antibodies). Scale bars for low-magnification are 100μm, for high-magnification are 50μm. Patients were classified into 2 groups according to the median protein expression of HERC3 (e). 250 patients were classified into 2 groups according the median expression of HERC3 or EIF5A2. Survival analysis were visualized by Kaplan-Meier plots and tested by log-rank (f). P<0.05 was identified as statistically significant. * represents P<0.05, ** represents P<0.01, and *** represents P<0.001.

Supplementary Fig. S7. EIF5A2 regulates the EMT/TGF-/Smad2/3 signal in CRC. Western blotting (a) indicated that EIF5A2 downregulation could independently increase the expression of E-cadherin and decrease the expression of N-cadherin and Vimentin in HCT116 cells and EIF5A2 overexpression could decrease the expression of E-cadherin and increase the expression of N-cadherin and Vimentin in SW620 cells. Moreover, EIF5A2 could regulate the TGF-/Smad2/3 signal. Overexpression EIF5A2 could enhance the expression of TGF-β1, p-Smad2 and p-Smad3 and downregulation EIF5A2 could inhibit the expression of TGF-β1, p-Smad2 and p-Smad3 (b).

Supplementary Fig. S8. IF indicated that EIF5A2 downregulation could independently increase the expression of E-cadherin and decrease the expression of N-cadherin and Vimentin in HCT116 cells and EIF5A2 overexpression could decrease the expression of E-cadherin and increase the expression of N-cadherin and Vimentin in SW620 cells. Scale bar for IF was 50μm.

Supplementary Fig. S9. IF indicated that EIF5A2 overexpression could rescue the effects of HERC overexpression on EMT HCT116 cells and EIF5A2 downregulation could also rescue the effects of HERC3 downregulation on EMT in SW620 cells. Experiments were conducted with indicated cell lines. Scale bar for IF was 50μm.

Supplementary Fig. S10. EIF5A2 increases the migration and invasion of CRC cells in vitro. a, Transwell assays indicated that EIF5A2 downregulation could inhibit the migration and invasion of HCT116 cells and EIF5A2 upregulation could increase the migration and invasion of SW620 cells. Scale bar for transwell assays was 100μm. b, Wound healing assays indicated that EIF5A2 downregulation could inhibit the migration of HCT116 cells while EIF5A2 upregulation increased the migration of SW620 cells. Scale bar for wound healing was 250μm.

Supplementary Fig. S11. Hematoxylin–eosin (HE) stain confirmed the metastasis in the liver formed by indicated cell lines in vivo experiments. Scale bars for low-magnification are 1000μm, for high-magnification are 100μm.

**Supplementary Table legend**

Supplementary Table S1. Peptides were identified by mass spectrometry analysis for the identification of ubiquitination modification sites.
