# Supplementary figures and images for: HERC3 regulates epithelial-mesenchymal transition by directly ubiquitination degradation EIF5A2 and inhibits metastasis of colorectal cancer
Source: Cell Death Dis. 2022 Jan 21;13(1):74. doi: 10.1038/s41419-022-04511-7 (PMC8782983; doi:10.1038/s41419-022-04511-7)

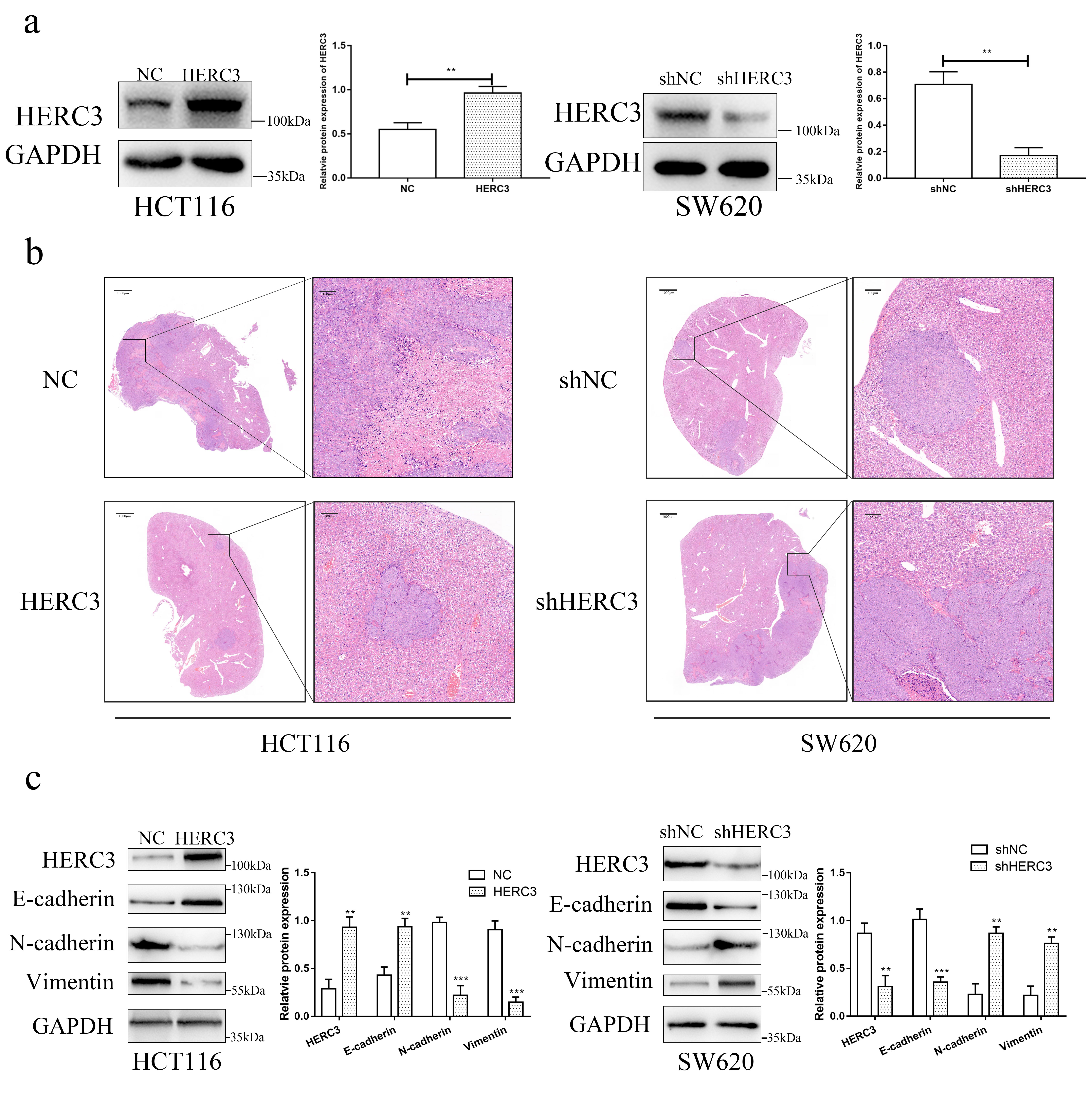

Supplement: Supplementary file 2 — Supplementary Fig. S1. [file 41419_2022_4511_MOESM2_ESM.jpg]

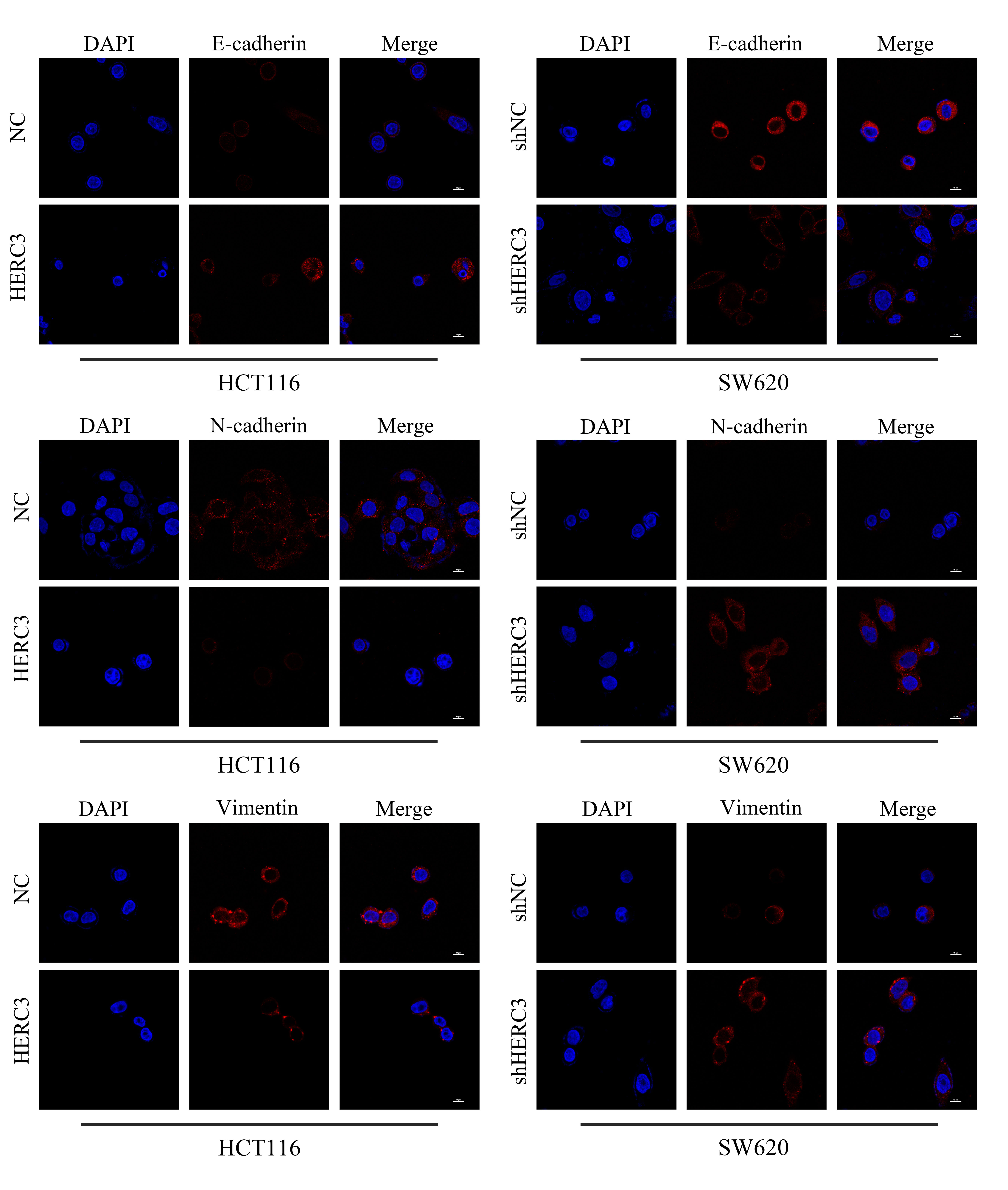

Supplement: Supplementary file 3 — Supplementary Fig. S2. [file 41419_2022_4511_MOESM3_ESM.jpg]

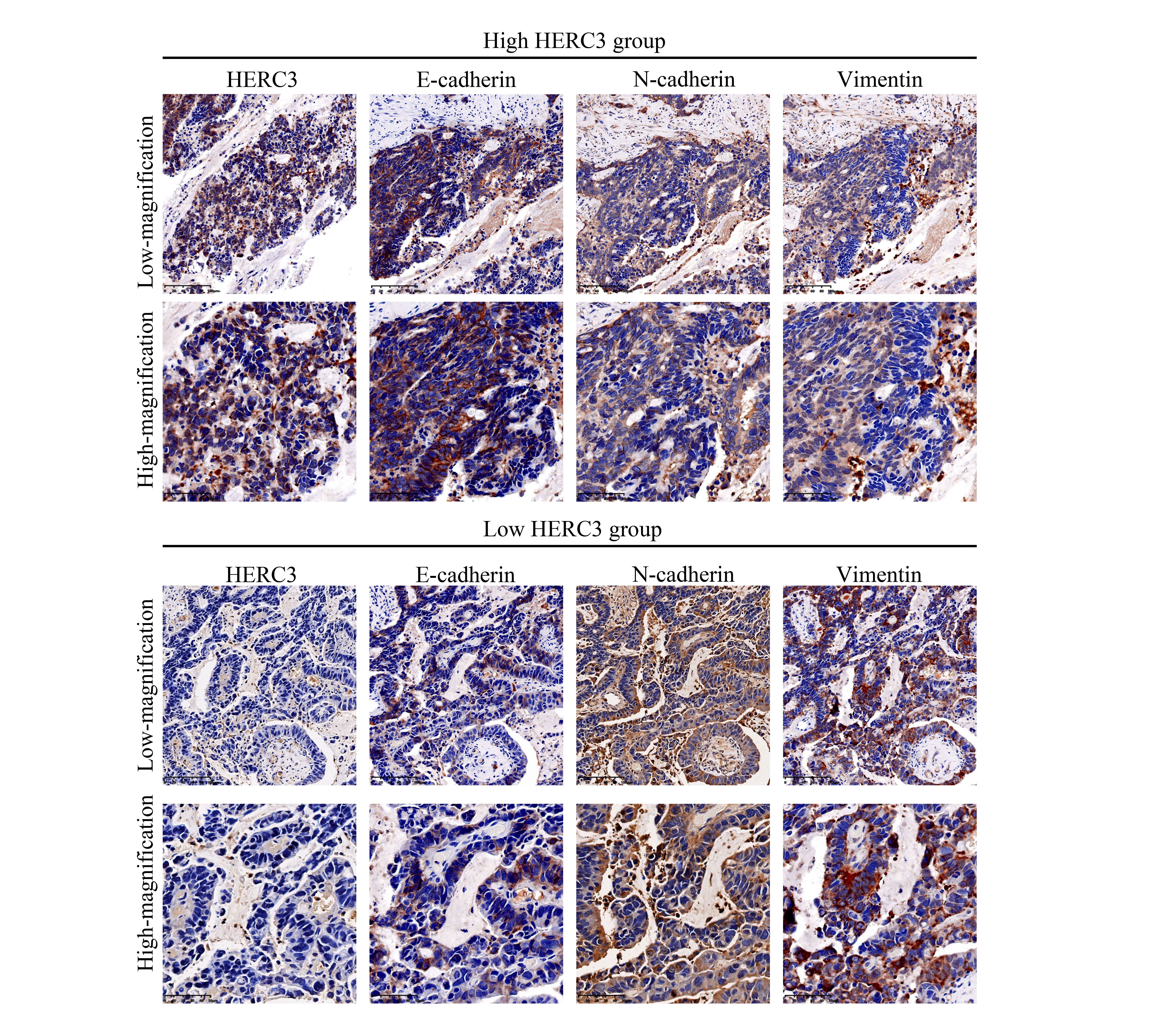

Supplement: Supplementary file 4 — Supplementary Fig. S3. [file 41419_2022_4511_MOESM4_ESM.jpg]

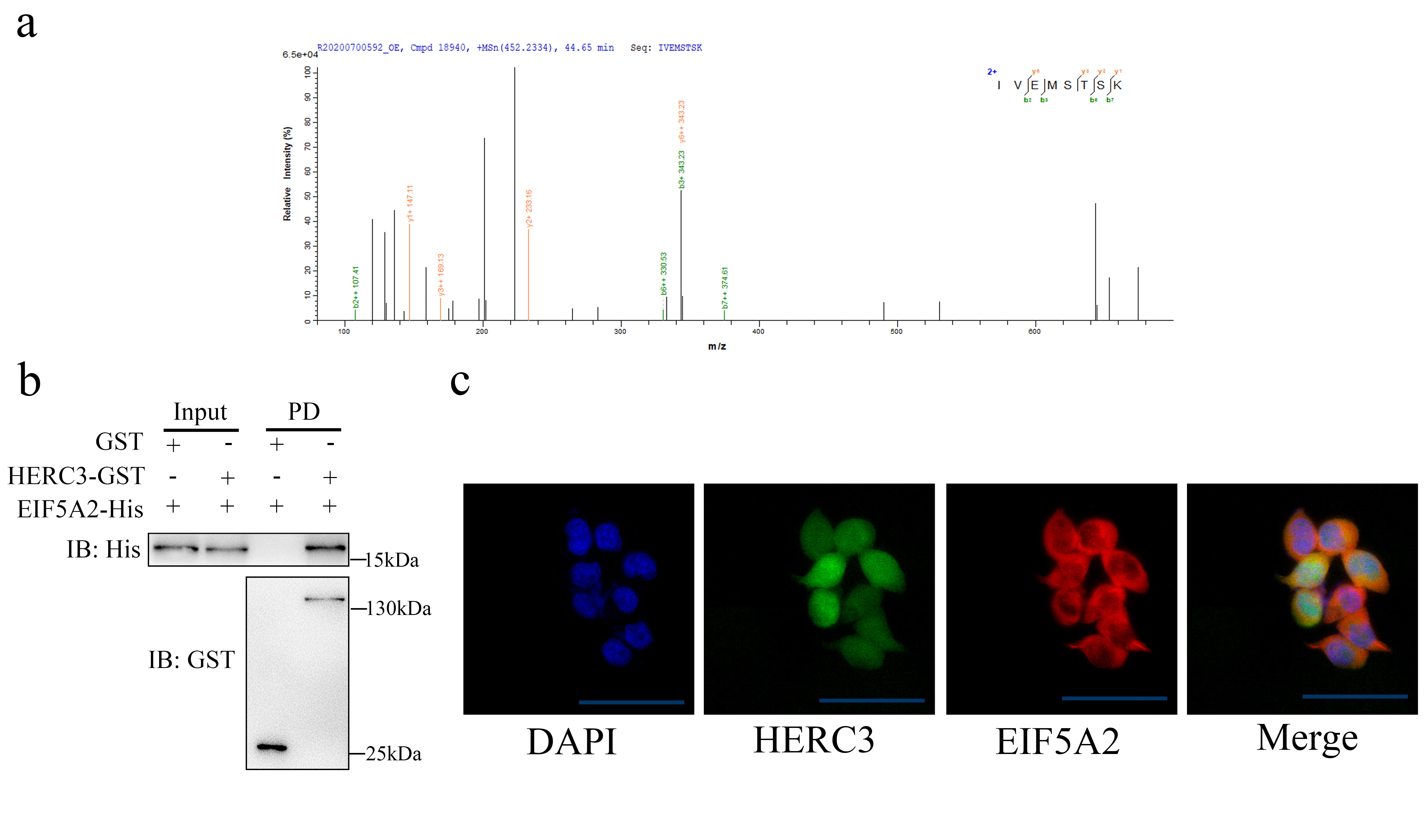

Supplement: Supplementary file 5 — Supplementary Fig. S4. [file 41419_2022_4511_MOESM5_ESM.jpg]

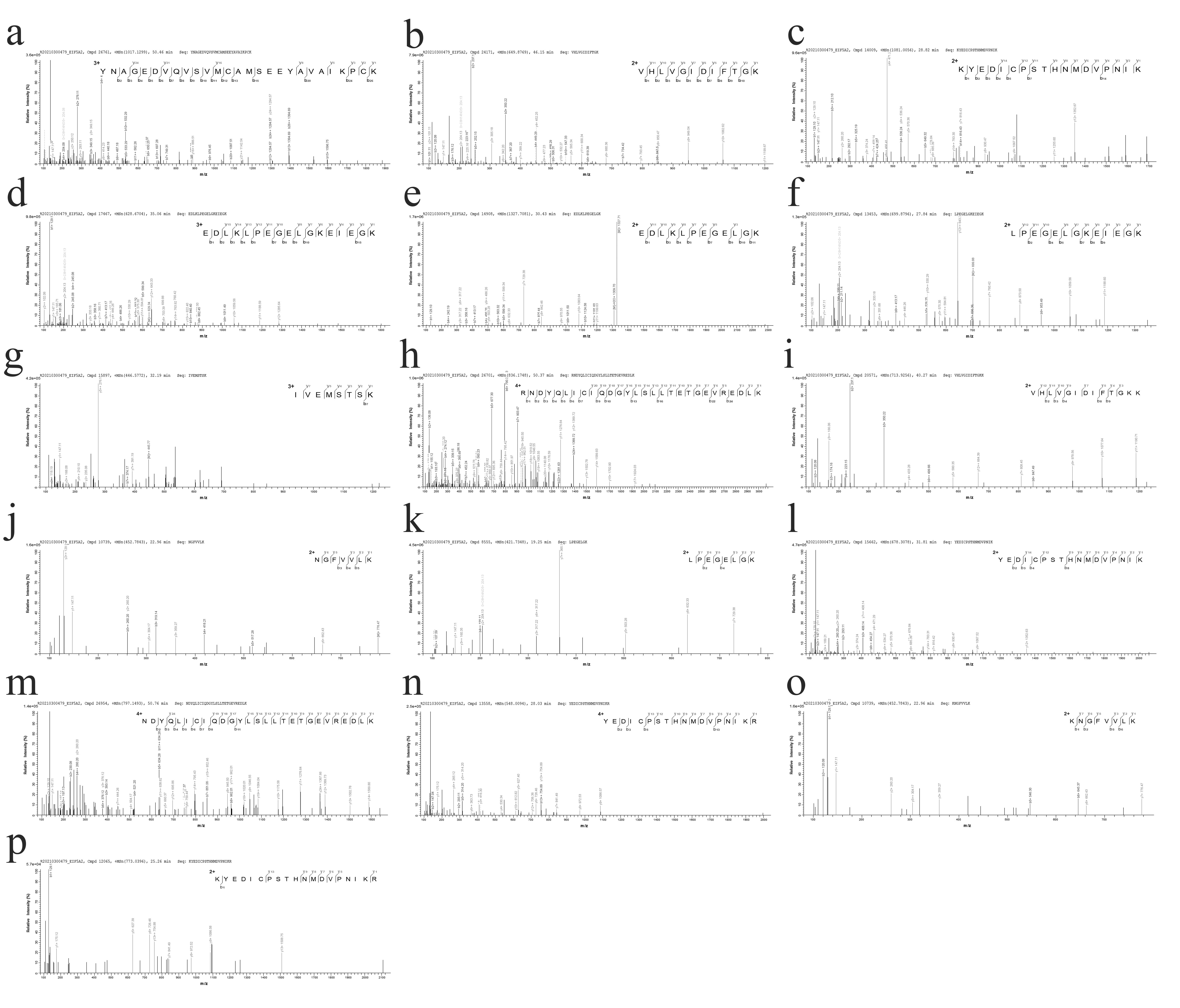

Supplement: Supplementary file 6 — Supplementary Fig. S5. [file 41419_2022_4511_MOESM6_ESM.jpg]

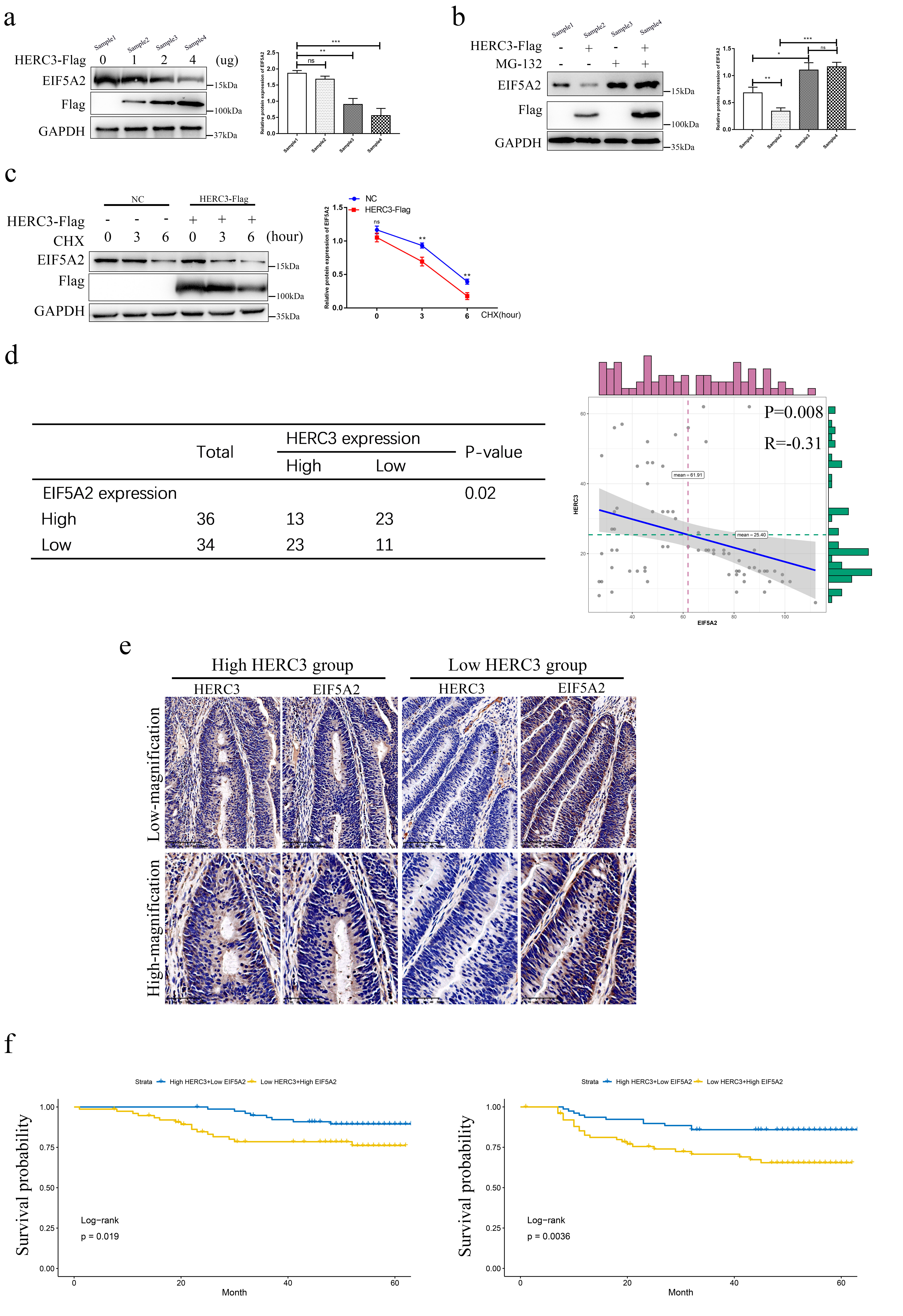

Supplement: Supplementary file 7 — Supplementary Fig. S6. [file 41419_2022_4511_MOESM7_ESM.jpg]

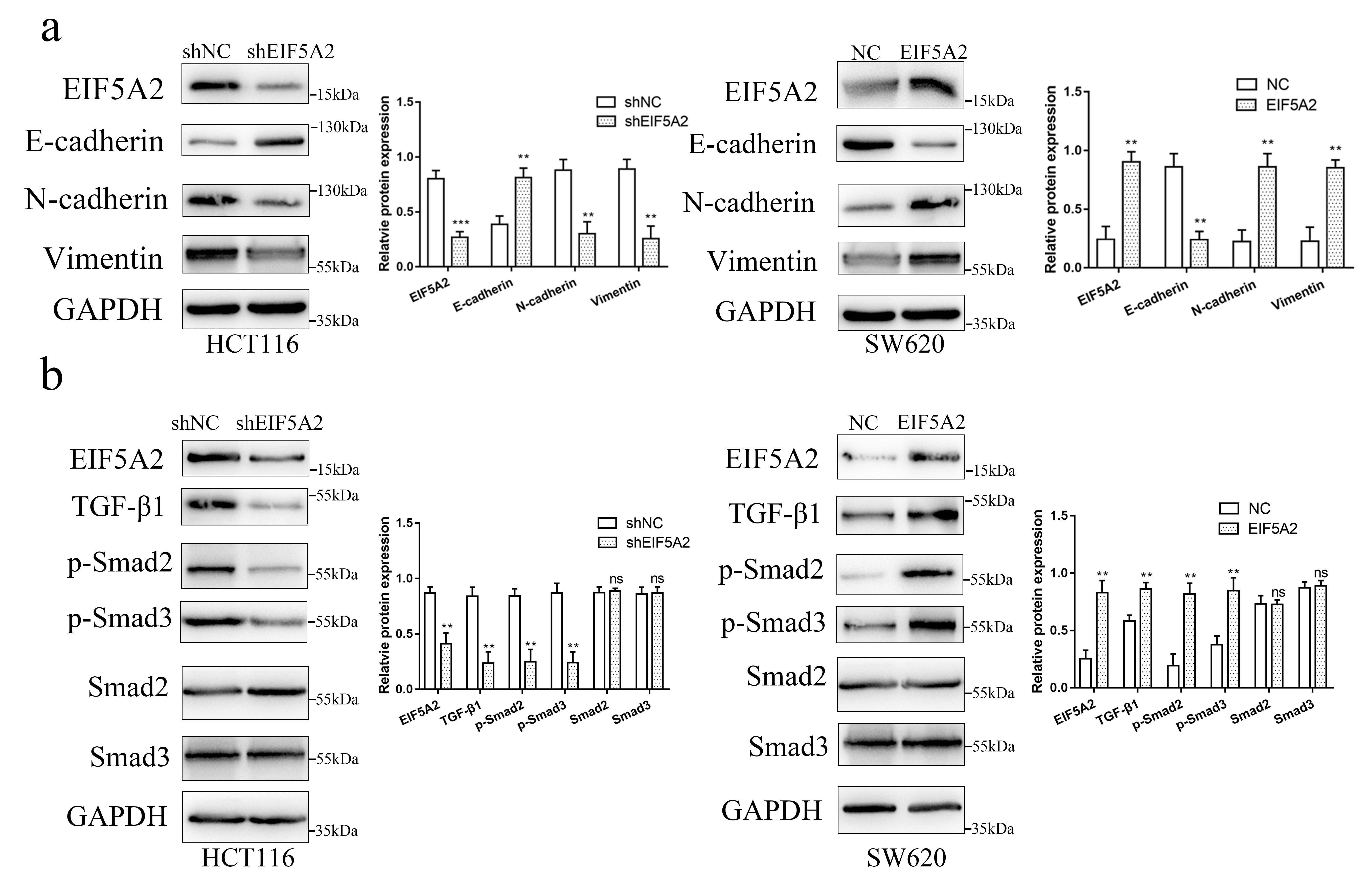

Supplement: Supplementary file 8 — Supplementary Fig. S7. [file 41419_2022_4511_MOESM8_ESM.jpg]

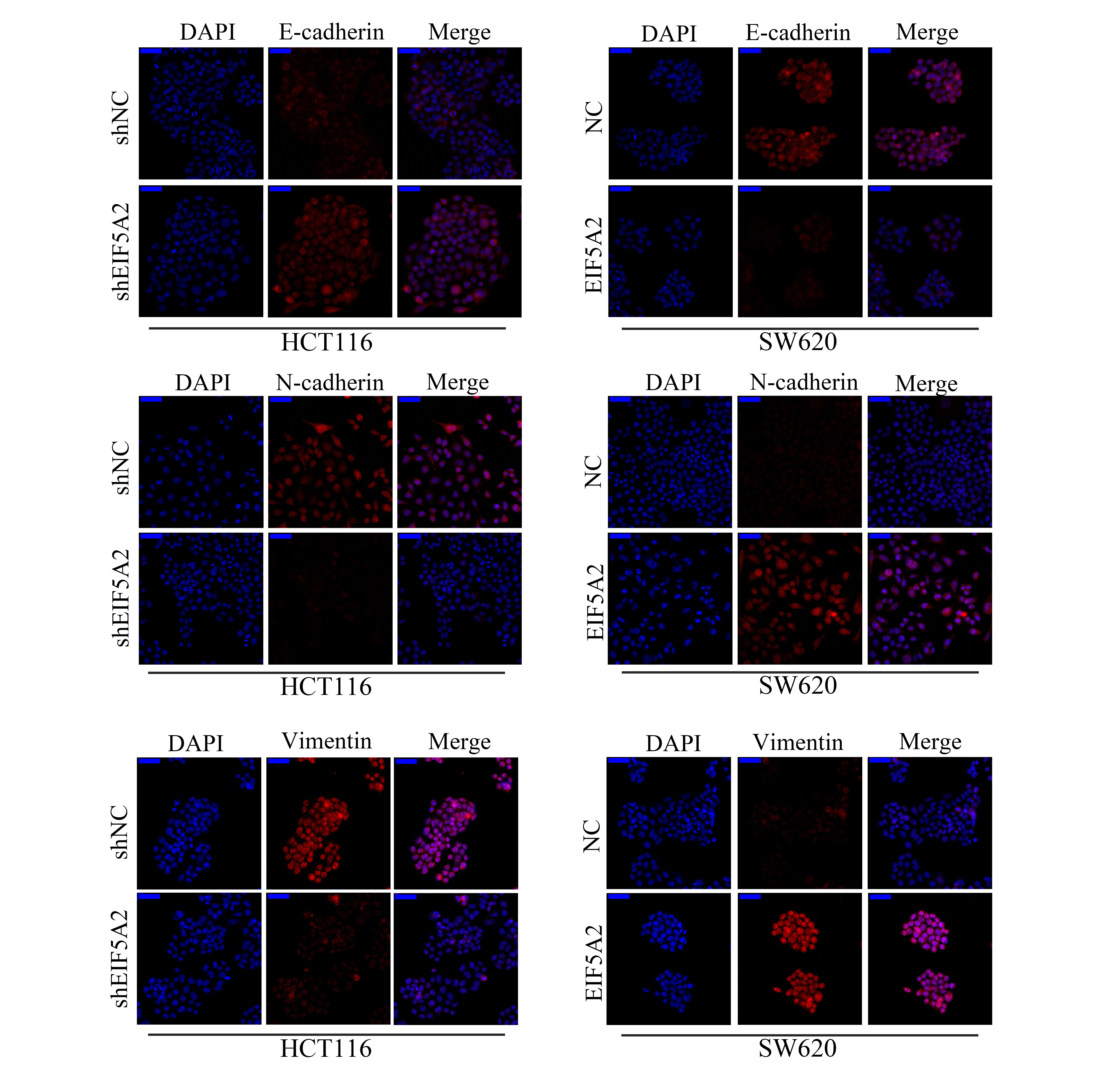

Supplement: Supplementary file 9 — Supplementary Fig. S8. [file 41419_2022_4511_MOESM9_ESM.jpg]

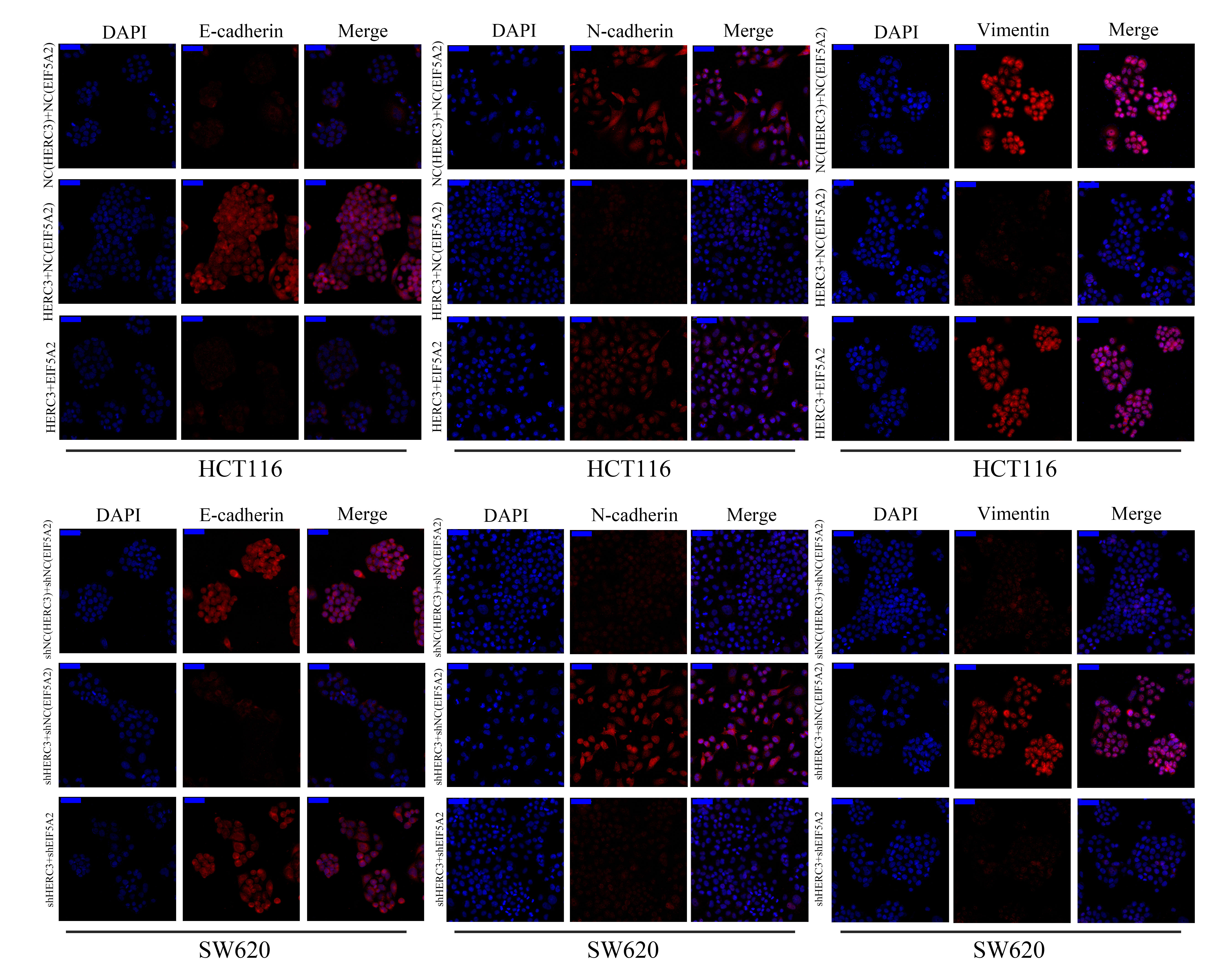

Supplement: Supplementary file 10 — Supplementary Fig. S9. [file 41419_2022_4511_MOESM10_ESM.jpg]

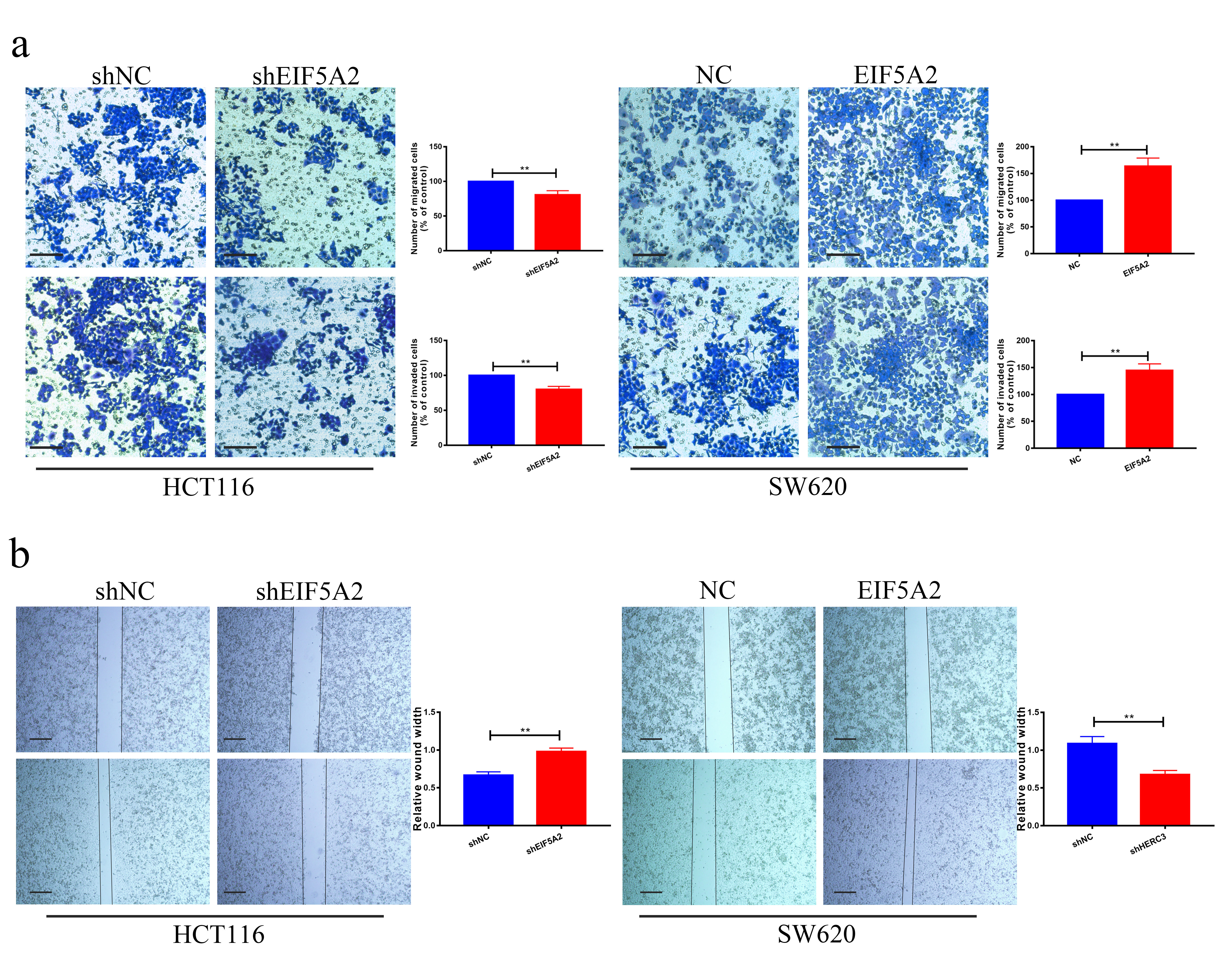

Supplement: Supplementary file 11 — Supplementary Fig. S10. [file 41419_2022_4511_MOESM11_ESM.jpg]

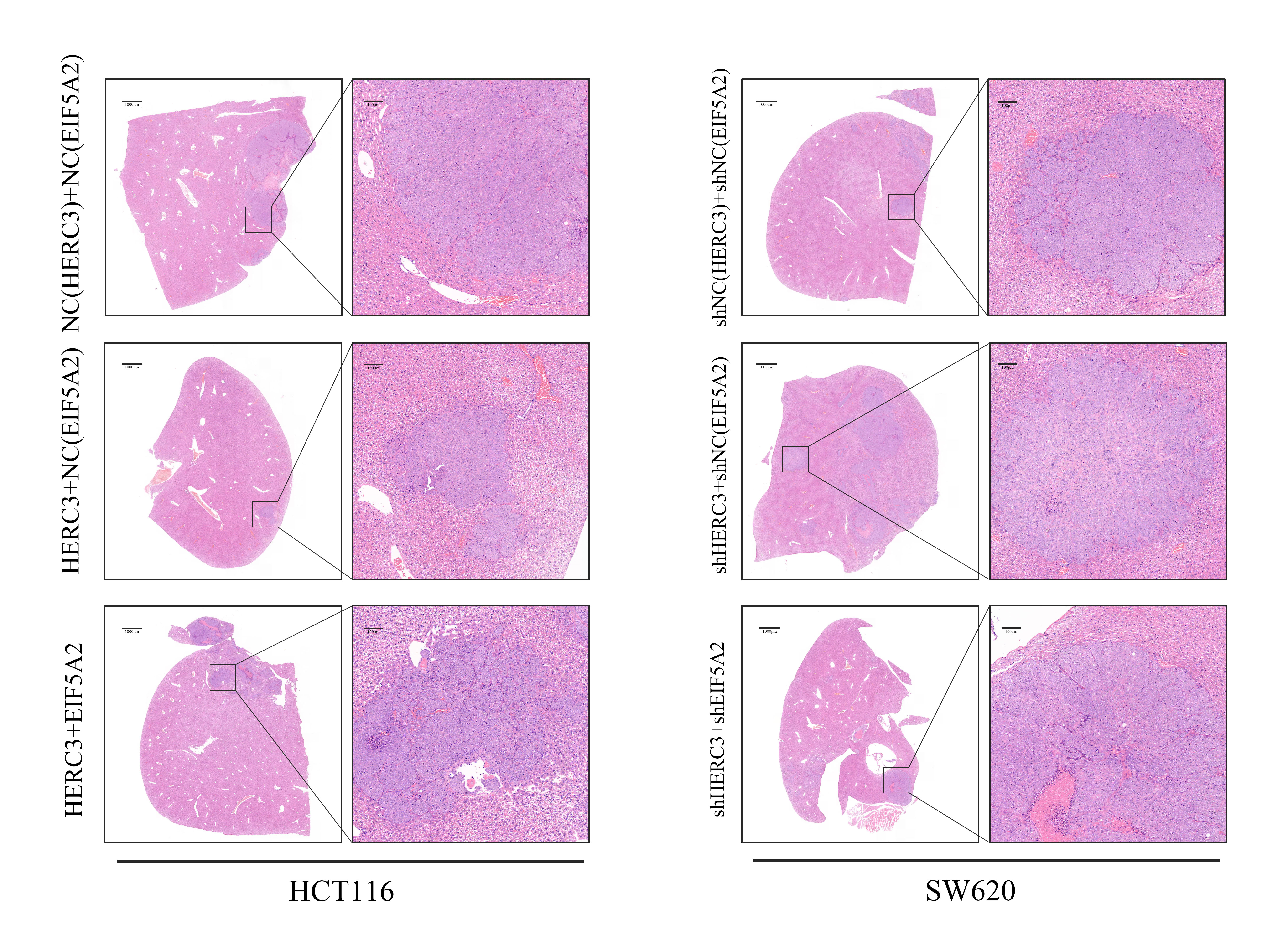

Supplement: Supplementary file 12 — Supplementary Fig. S11. [file 41419_2022_4511_MOESM12_ESM.jpg]
