# Supplementary material for: HERC3 regulates epithelial-mesenchymal transition by directly ubiquitination degradation EIF5A2 and inhibits metastasis of colorectal cancer
Source: Cell Death Dis. 2022 Jan 21;13(1):74. doi: 10.1038/s41419-022-04511-7 (PMC8782983; doi:10.1038/s41419-022-04511-7)
Supplement: Supplementary file 15 — Response of the change of the authors [file 41419_2022_4511_MOESM15_ESM.pdf]

163 网易免费邮 mail.163.com | zsyyxjm@163.com | Settings | Download App | Feedback | Join Member

Home | Contacts | Application... | Inbox | Re:Confri...

Check | Compose

Inbox, Red Flag, To-do, Auto-labeled, Starred, Drafts, Sent, Subscription, Other 4 folders, Labels, Pop folder, File Center, Mailbox attachment, Office templates NEW

Re:Confrim of the change of the authors of our manusript (CDDIS-21-3098RR)

From: Zhiyuan Zhang<635920161@qq.com>

To: zsyyxjm<zsyyxjm@163.com> coryhu<coryhu@sina.com> zsyyliuyu<zsyyliuyu@126.com> zsyyhgd<zsyyhgd@163.com> zsyylvya<zsyylvya@163.com> ↓ there are still 2contacts Save AllTo

Date: 2021-12-24 08:15

Translate into Chinese

Yes, thanks for you mail!

---------------- Original ----------------

From: "zsyyxjm" <zsyyxjm@163.com>;
Date: Fri, Dec 24, 2021 08:14 AM
To: "coryhu"<coryhu@sina.com>;"zsyyliuyu"<zsyyliuyu@126.com>;"zsyyhgd"<zsyyhgd@163.com>;"zsyylvya"<zsyylvya@163.com>;"zsyyfqy"<zsyyfqy@163.com>;"zsyyniuzc"<zsyyniuzc@163.com>;"Zhiyuan Zhang"<635920161@qq.com>;
Subject: Confrim of the change of the authors of our manusript (CDDIS-21-3098RR)

Dear colleagues,
It's a mail to confirm that you all agree to add Dr. Yu Liu as a co-author in our paper entitled "HERC3 regulates epithelial-mesenchymal transition by directly ubiquitination degradation EIF5A2 and inhibits metastasis of colorectal cancer.
" submitted to *Cell Death & Disease* (CDDIS-21-3098RR). The updated order of authors is: Zhiyuan Zhang , Guodong He, Yang Lv, Yu Liu, Zhengchuan Niu, Qingyang Feng, Ronggui Hu, Jianmin Xu. Please reply "Yes" to confirm this change via emial. Thanks a lot.
Best regards,
Prof. Jianmin Xu, zsyyxjm@163.com.
Department of General Surgery, Zhongshan Hospital, Fudan University, 200030 Shanghai, China.

Quick Reply to: Zhiyuan Zhang,coryhu ,zsyyliuyu ,zsyyhgd ,zsyylvya ,zsyyfqy ,zsyyniuzc

---

163 网易免费邮 mail.163.com | zsyyxjm@163.com | Settings | Download App | Feedback | Join Member

Home | Contacts | Application... | Inbox | Re:Confrim ... | Re:Confrim ... | Re:Confri...

Re:Confrim of the change of the authors of our manusript (CDDIS-21-3098RR)

From: zsyyhgd<zsyyhgd@163.com>

To: zsyyxjm<zsyyxjm@163.com>

Date: 2021-12-24 13:31

yes

At 2021-12-24 08:14:30, "zsyyxjm" <zsyyxjm@163.com> wrote:

- Hide cited text -

Dear colleagues,
It's a mail to confirm that you all agree to add Dr. Yu Liu as a co-author in our paper entitled "HERC3 regulates epithelial-mesenchymal transition by directly ubiquitination degradation EIF5A2 and inhibits metastasis of colorectal cancer.
" submitted to *Cell Death & Disease* (CDDIS-21-3098RR). The updated order of authors is: Zhiyuan Zhang , Guodong He, Yang Lv, Yu Liu, Zhengchuan Niu, Qingyang Feng, Ronggui Hu, Jianmin Xu. Please reply "Yes" to confirm this change via emial. Thanks a lot.
Best regards,
Prof. Jianmin Xu, zsyyxjm@163.com.
Department of General Surgery, Zhongshan Hospital, Fudan University, 200030 Shanghai, China.

Quick Reply to: zsyyhgd

javascript:void(0);

---

163 网易免费邮 mail.163.com | zsyyxjm@163.com | Settings | Download App | Feedback | Join Member

Home | Contacts | Application... | Inbox | Re:Confrim ... | Re:Confri...

Re:Confrim of the change of the authors of our manusript (CDDIS-21-3098RR)

From: zsyylvya<zsyylvya@163.com>

To: zsyyxjm<zsyyxjm@163.com>

Date: 2021-12-24 08:17

Yes. It's OK.

At 2021-12-24 08:14:30, "zsyyxjm" <zsyyxjm@163.com> wrote:

- Hide cited text -

Dear colleagues,
It's a mail to confirm that you all agree to add Dr. Yu Liu as a co-author in our paper entitled "HERC3 regulates epithelial-mesenchymal transition by directly ubiquitination degradation EIF5A2 and inhibits metastasis of colorectal cancer.
" submitted to *Cell Death & Disease* (CDDIS-21-3098RR). The updated order of authors is: Zhiyuan Zhang , Guodong He, Yang Lv, Yu Liu, Zhengchuan Niu, Qingyang Feng, Ronggui Hu, Jianmin Xu. Please reply "Yes" to confirm this change via emial. Thanks a lot.
Best regards,
Prof. Jianmin Xu, zsyyxjm@163.com.
Department of General Surgery, Zhongshan Hospital, Fudan University, 200030 Shanghai, China.

Quick Reply to: zsyylvya

Re:Confrim of the change of the authors of our manusript (CDDIS-21-3098RR)

From: zsyyliuyu <zsyyliuyu@126.com>

To: zsyyxjm <zsyyxjm@163.com>

Date: 2021-12-24 12:29

yes

At 2021-12-24 08:14:30, "zsyyxjm" <zsyyxjm@163.com> wrote:

- Hide cited text -

Dear colleagues,

It's a mail to confirm that you all agree to add Dr. Yu Liu as a co-author in our paper entitled "HERC3 regulates epithelial-mesenchymal transition by directly ubiquitination degradation EIF5A2 and inhibits metastasis of colorectal cancer.

" submitted to *Cell Death & Disease* (CDDIS-21-3098RR). The updated order of authors is: Zhiyuan Zhang , Guodong He, Yang Lv, Yu Liu, Zhengchuan Niu, Qingyang Feng, Ronggui Hu, Jianmin Xu. Please reply "Yes" to confirm this change via emial. Thanks a lot.

Best regards,

Prof. Jianmin Xu, zsyyxjm@163.com.

Department of General Surgery, Zhongshan Hospital, Fudan University, 200030 Shanghai, China.

Re: Confrim of the change of the authors of our manusript(CDDIS-21-3098RR)

From: Zhengchuan Niu <niuzhch@qq.com>

To: zsyyxjm <zsyyxjm@163.com>

Date: 2021-12-26 11:11

yes

---Original---

From: "zsyyxjm"<zsyyxjm@163.com>
Date: Fri, Dec 24, 2021 08:27 AM
To: "niuzhch"<niuzhch@qq.com>;
Subject: Confrim of the change of the authors of our manusript(CDDIS-21-3098RR)

Dear colleagues,

It's a mail to confirm that you all agree to add Dr. Yu Liu as a co-author in our paper entitled "HERC3 regulates epithelial-mesenchymal transition by directly ubiquitination degradation EIF5A2 and inhibits metastasis of colorectal cancer.

" submitted to *Cell Death & Disease* (CDDIS-21-3098RR). The updated order of authors is: Zhiyuan Zhang , Guodong He, Yang Lv, Yu Liu, Zhengchuan Niu, Qingyang Feng, Ronggui Hu, Jianmin Xu. Please reply "Yes" to confirm this change via emial. Thanks a lot.

Best regards,

Prof. Jianmin Xu, zsyyxjm@163.com.

Department of General Surgery, Zhongshan Hospital, Fudan University, 200030 Shanghai, China.

Re:Confrim of the change of the authors of our manusript (CDDIS-21-3098RR)

From: zsyyfqy <zsyyfqy@163.com>

To: zsyyxjm <zsyyxjm@163.com>

Date: 2021-12-24 12:21

Yes

At 2021-12-24 08:14:30, "zsyyxjm" <zsyyxjm@163.com> wrote:

- Hide cited text -

Dear colleagues,

It's a mail to confirm that you all agree to add Dr. Yu Liu as a co-author in our paper entitled "HERC3 regulates epithelial-mesenchymal transition by directly ubiquitination degradation EIF5A2 and inhibits metastasis of colorectal cancer.

" submitted to *Cell Death & Disease* (CDDIS-21-3098RR). The updated order of authors is: Zhiyuan Zhang , Guodong He, Yang Lv, Yu Liu, Zhengchuan Niu, Qingyang Feng, Ronggui Hu, Jianmin Xu. Please reply "Yes" to confirm this change via emial. Thanks a lot.

Best regards,

Prof. Jianmin Xu, zsyyxjm@163.com.

Department of General Surgery, Zhongshan Hospital, Fudan University, 200030 Shanghai, China.

回复：Confrim of the change of the authors of our manusript (CDDIS-21-3098RR)

Reading Optimization | Concise Information

From: coryhu<coryhu@sina.com>

To: zsyyxjm<zsyyxjm@163.com>

Date: 2021-12-24 08:19

Translate into Chinese

Yes.

----- 原始邮件 -----
发件人：zsyyxjm <zsyyxjm@163.com>
收件人：coryhu@sina.com, zsyyliuyu@126.com, zsyyhgd@163.com, zsyylvya@163.com, zsyyfqy@163.com, zsyyniuzc@163.com, "635920161@qq.com" <635920161@qq.com>
主题：Confrim of the change of the authors of our manusript (CDDIS-21-3098RR)
日期：2021年12月24日 08点14分

Dear colleagues,

It's a mail to confirm that you all agree to add Dr. Yu Liu as a co-author in our paper entitled "HERC3 regulates epithelial-mesenchymal transition by directly ubiquitination degradation EIF5A2 and inhibits metastasis of colorectal cancer.

" submitted to *Cell Death & Disease* (CDDIS-21-3098RR). The updated order of authors is: Zhiyuan Zhang , Guodong He, Yang Lv, Yu Liu, Zhengchuan Niu, Qingyang Feng, Ronggui Hu, Jianmin Xu. Please reply "Yes" to confirm this change via emial. Thanks a lot.

Best regards,

Prof. Jianmin Xu, zsyyxjm@163.com.
Department of General Surgery, Zhongshan Hospital, Fudan University, 200030 Shanghai, China.

Quick Reply to: coryhu
